# Supplementary material for: TERT Expression in Wilms Tumor Is Regulated by Promoter Mutation or Hypermethylation, WT1, and N-MYC
Source: Cancers (Basel). 2022 Mar 25;14(7):1655. doi: 10.3390/cancers14071655 (PMC8996936; doi:10.3390/cancers14071655)
Supplement: Supplementary file 1 [file cancers-14-01655-s001.zip › Supplementary Data File S3.pdf]

Original Labeled Western Blot Images:

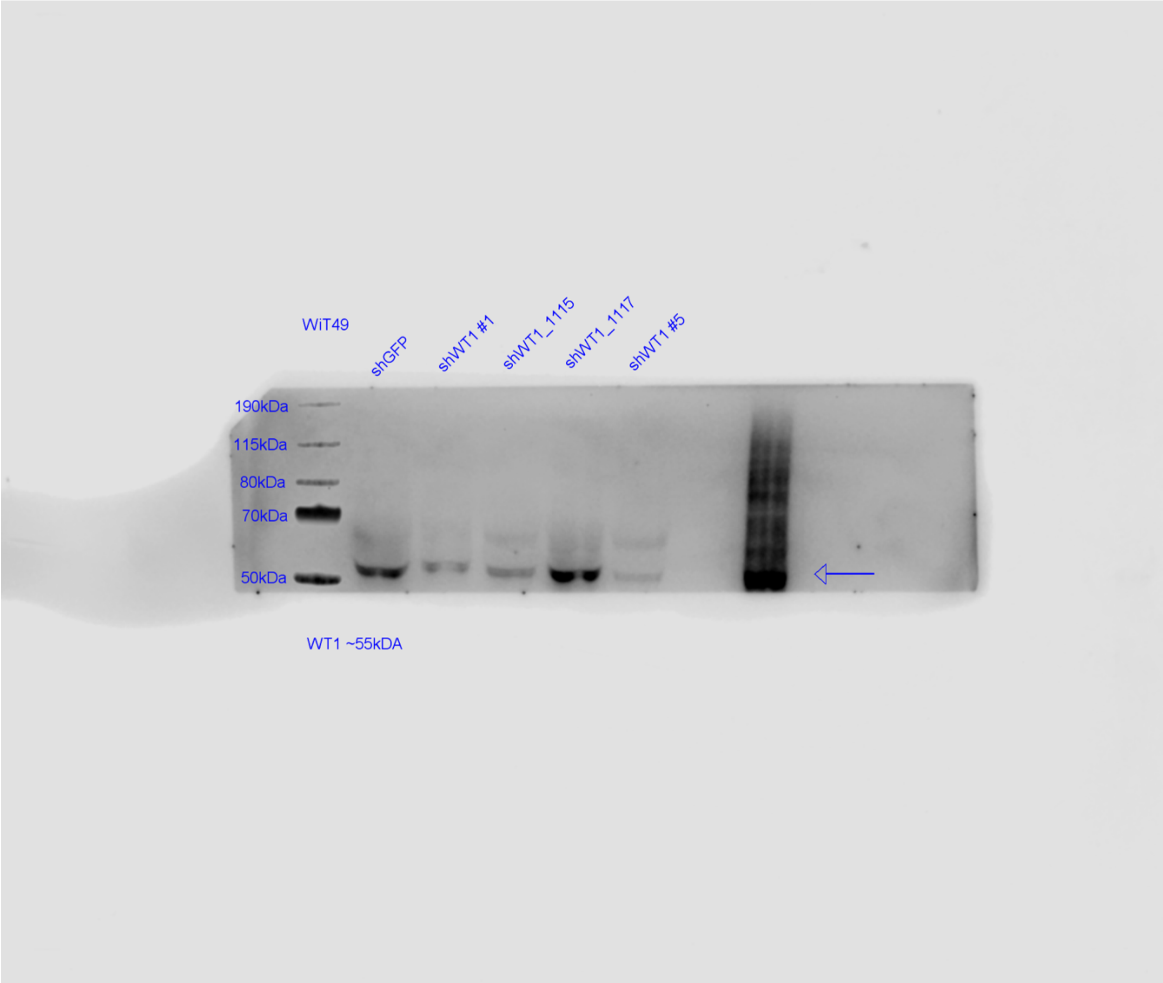

Figure 4a: WT1

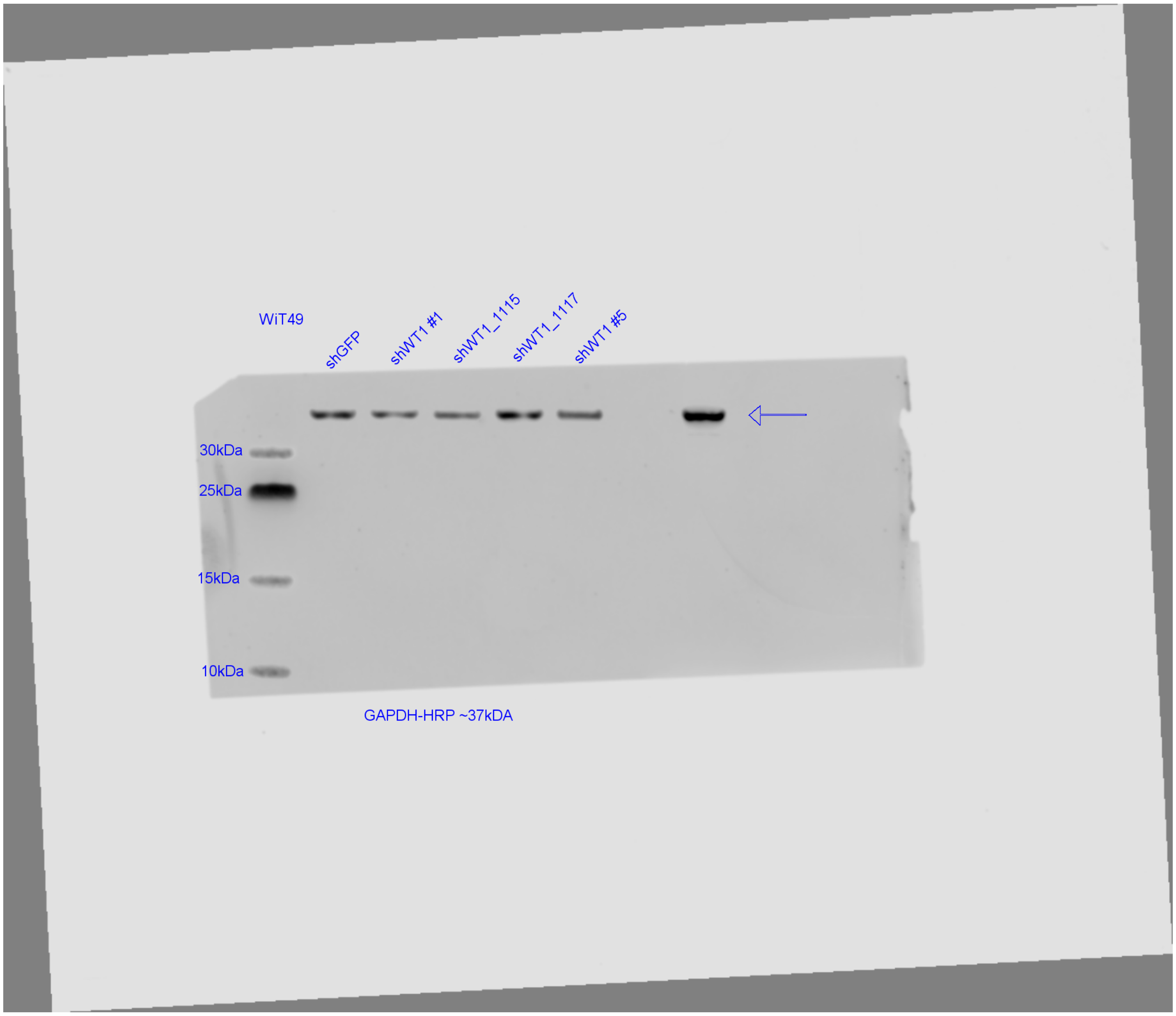

Figure 4a: GAPDH-HRP

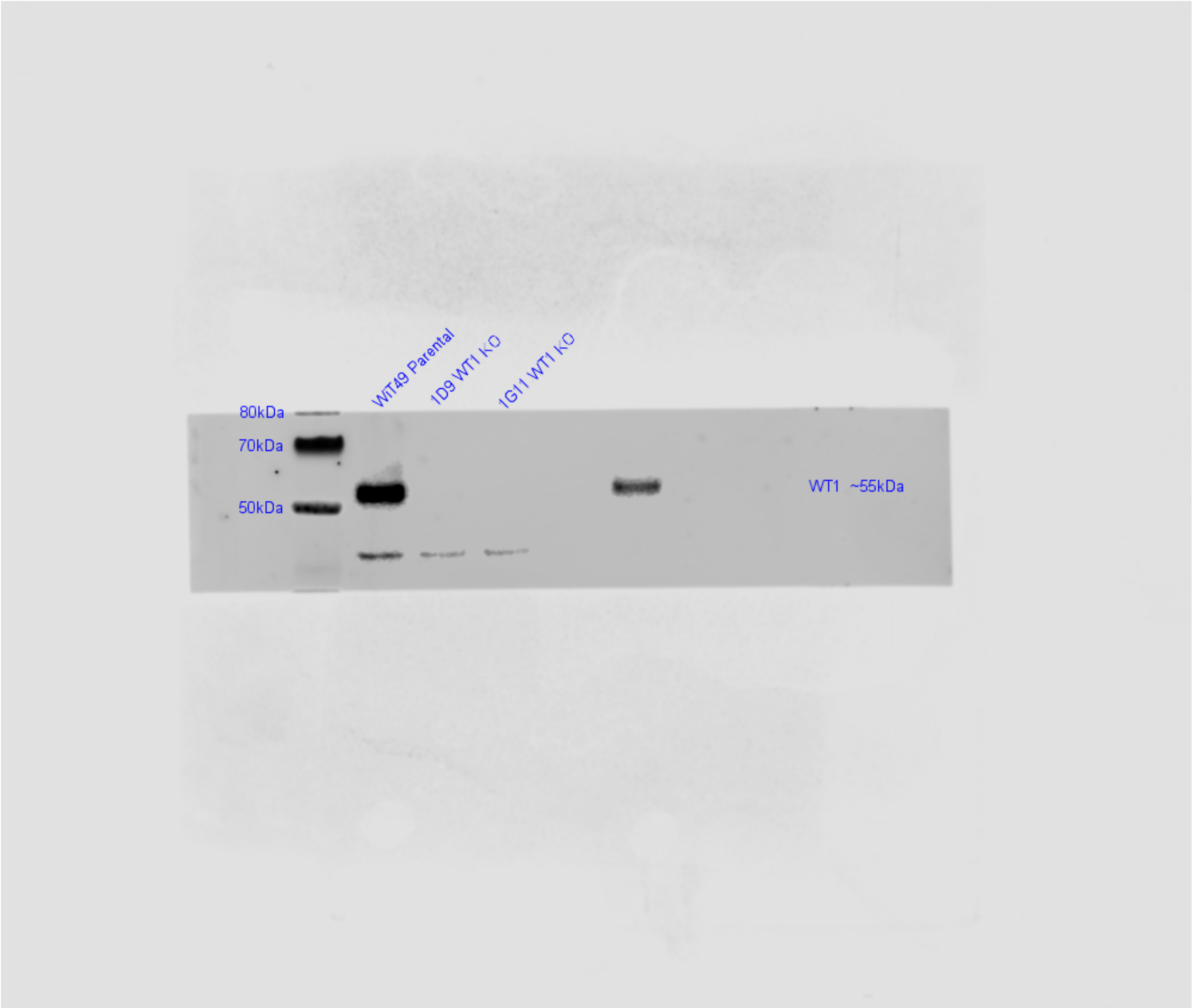

Figure 4f: WiT49 cells WT1

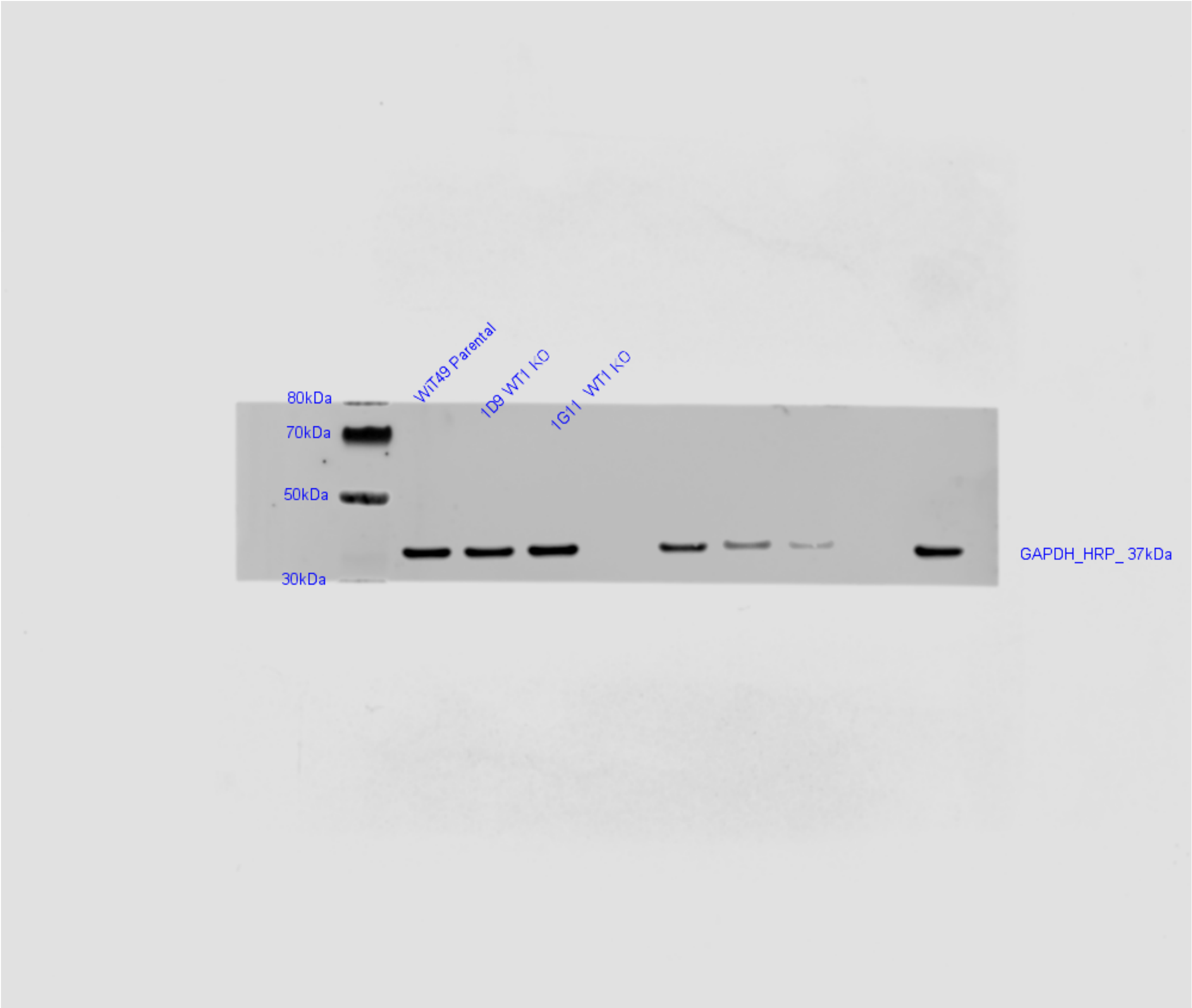

Figure 4f: WiT49 cells GAPDH-HRP

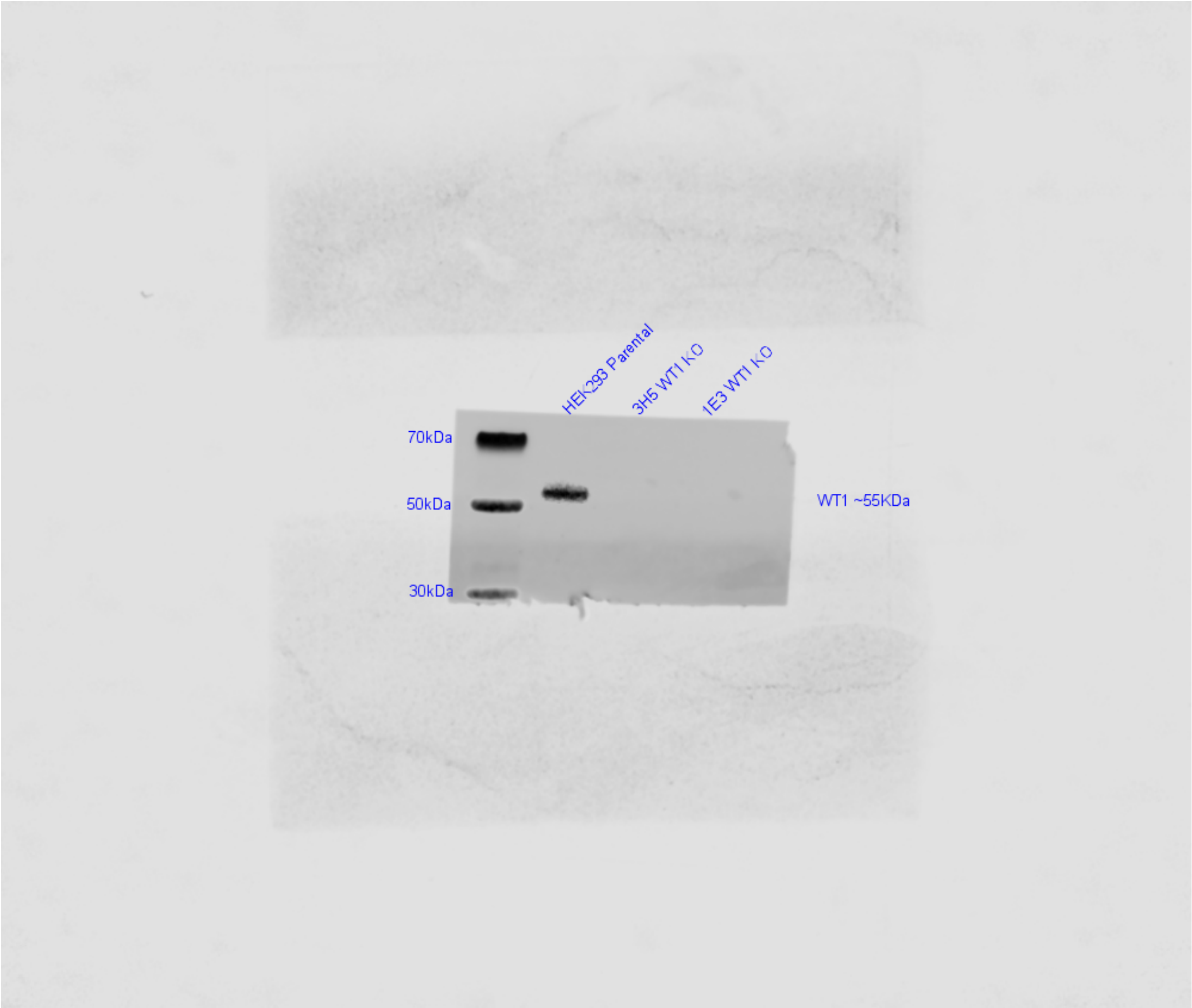

Figure 4f: HEK293 cells WT1

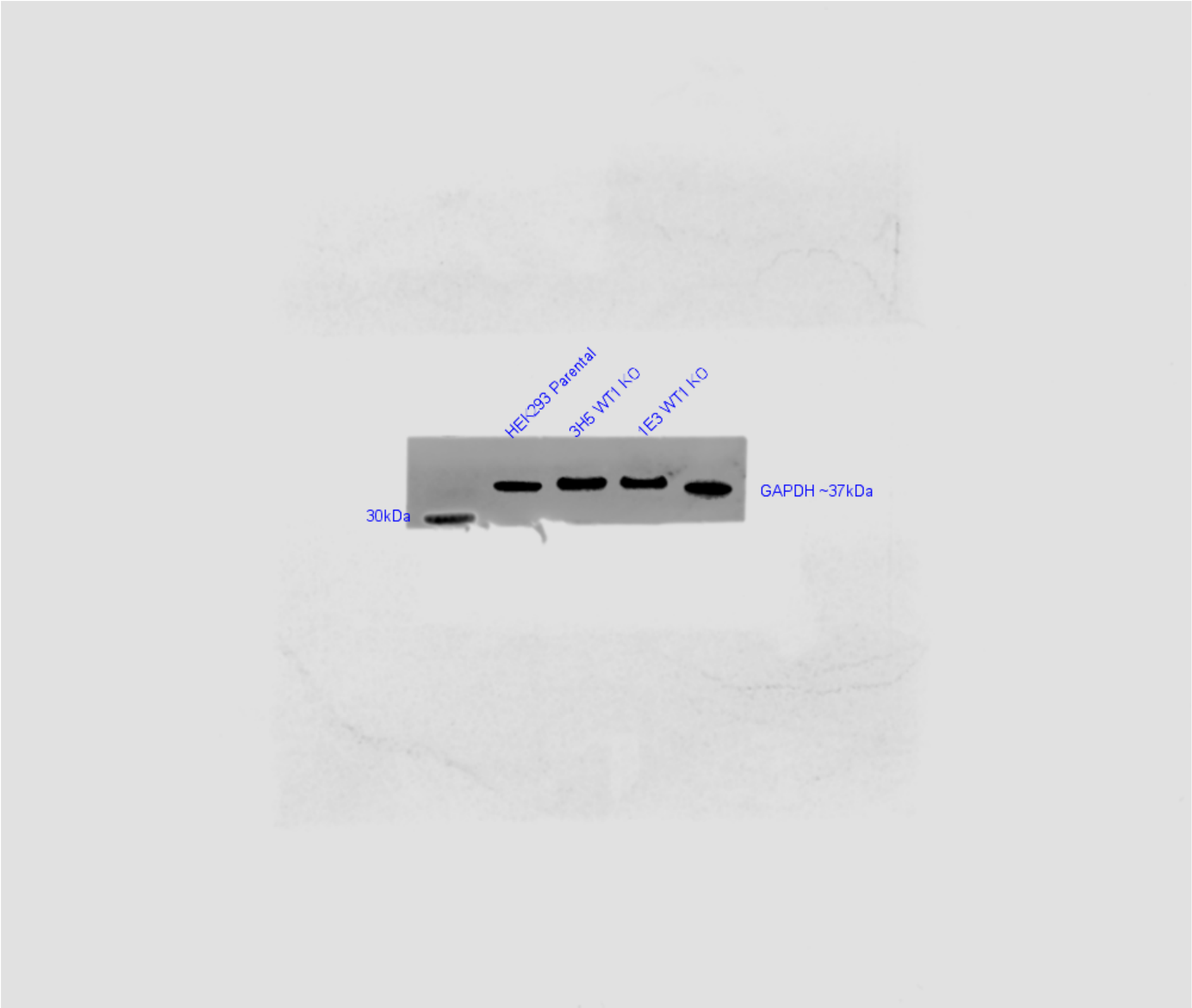

Figure 4f: HEK293 cells GAPDH-HRP

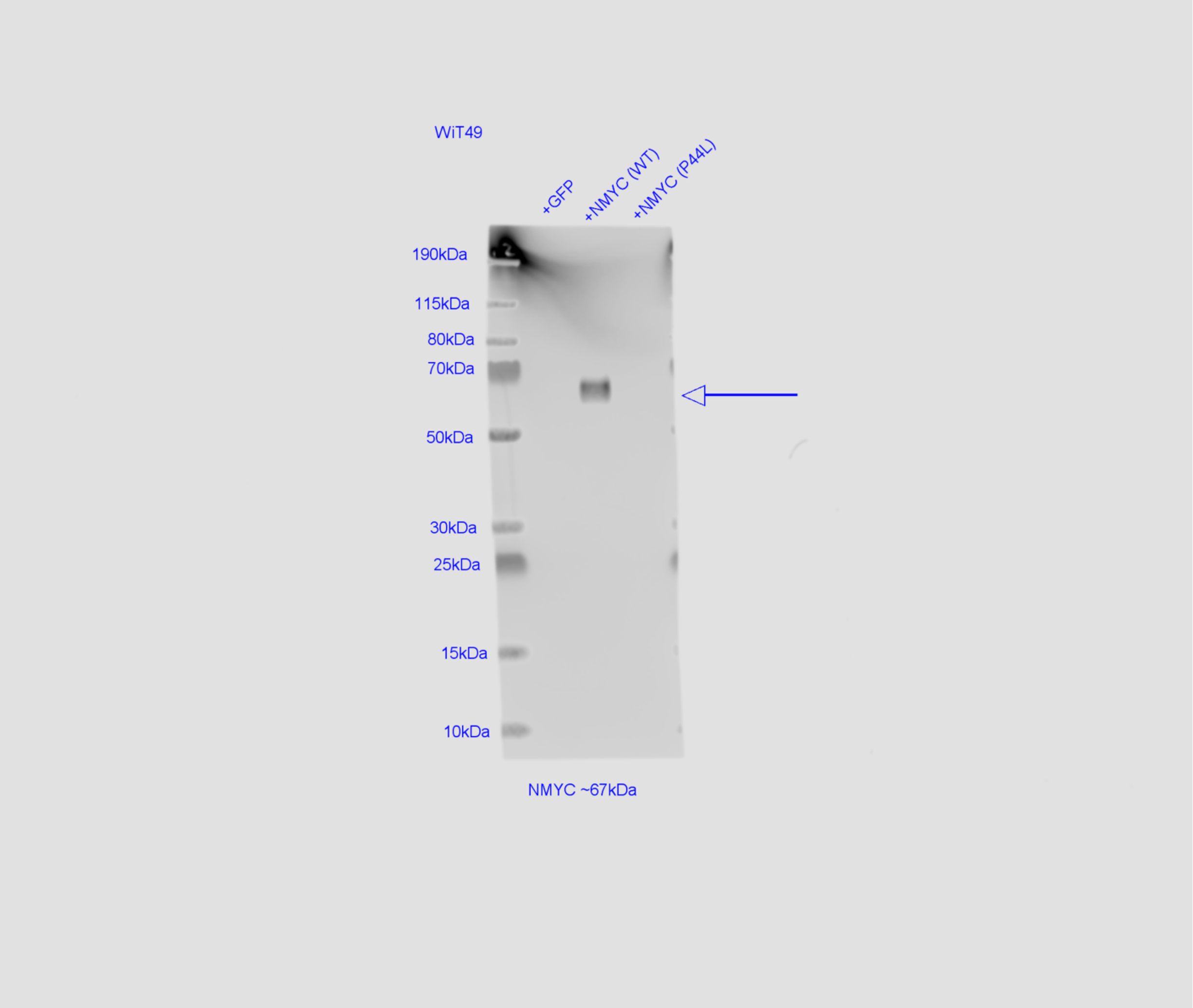

Figure 6c: N-MYC (N-terminus) short exposure

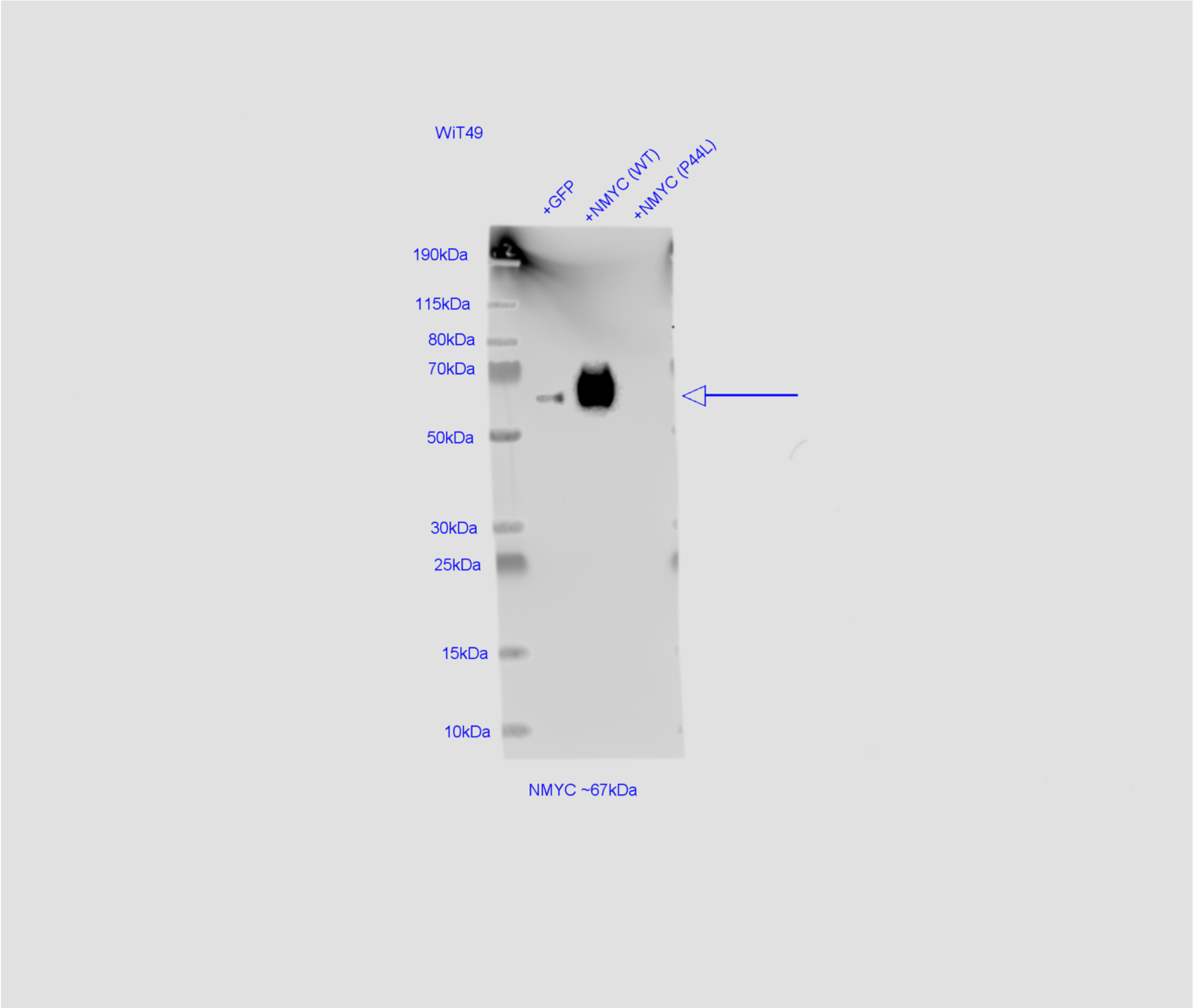

Figure 6c: N-MYC (N-terminus) long exposure

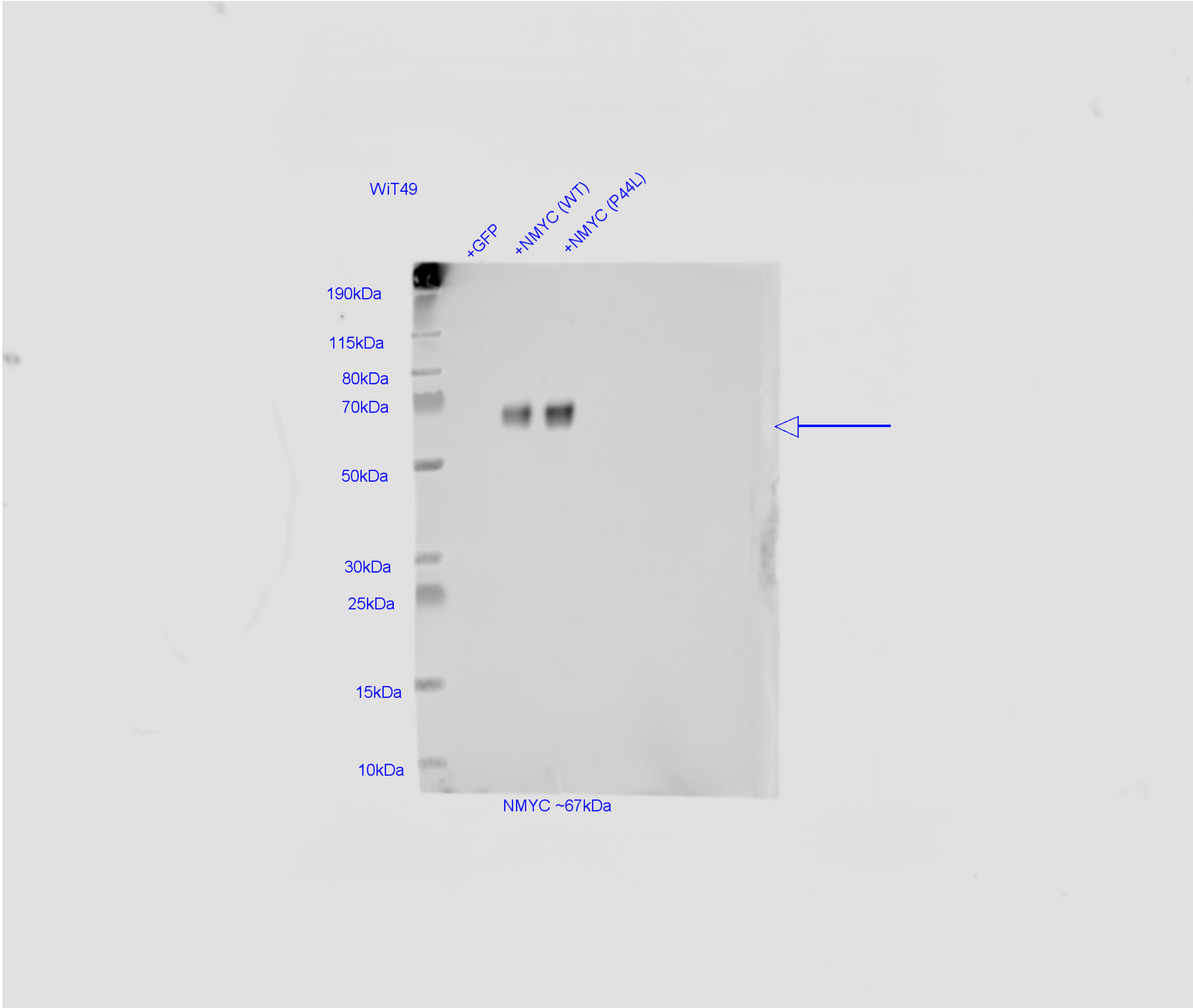

Figure 6c: N-MYC (C-terminus) short exposure

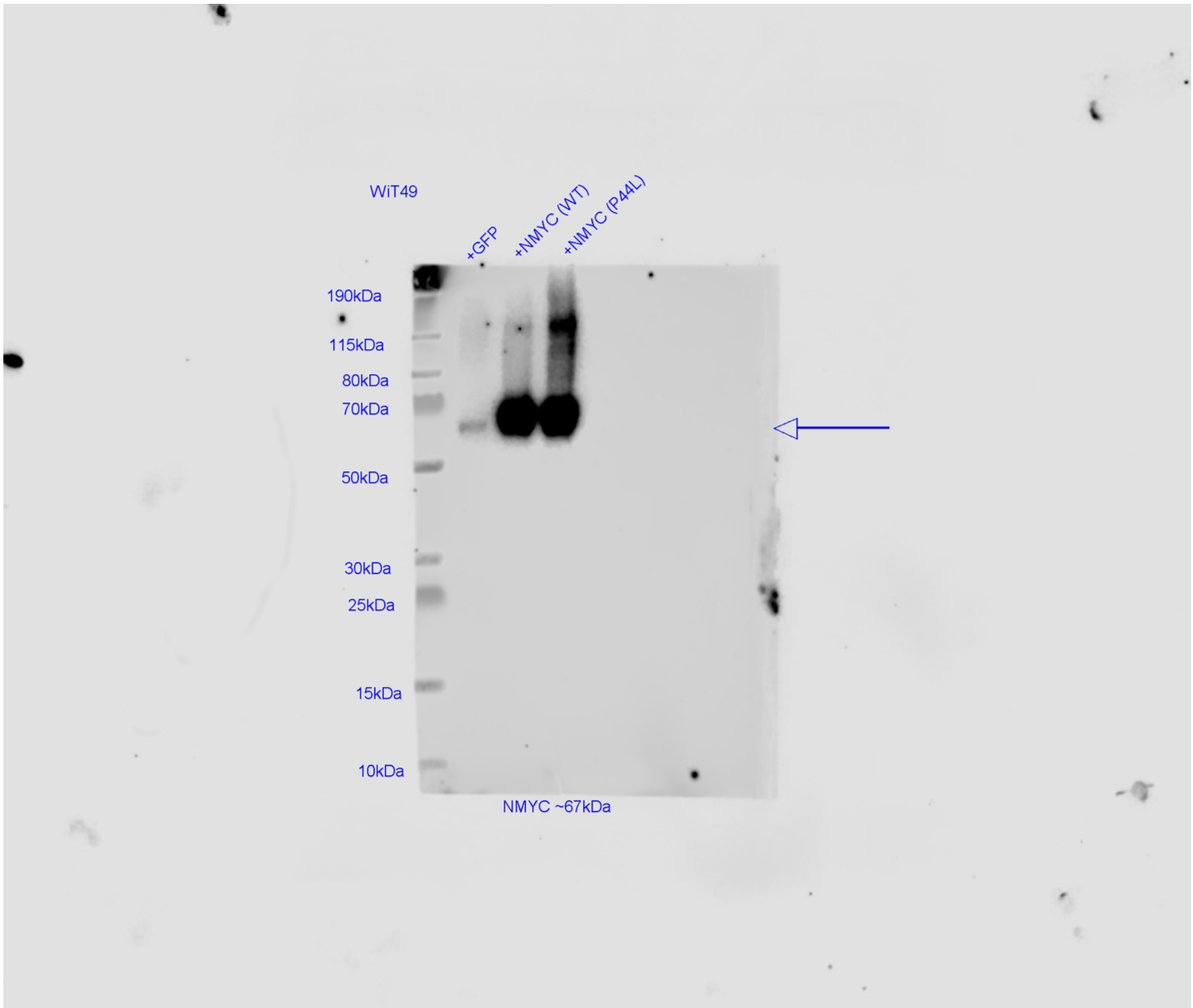

Figure 6c: N-MYC (C-terminus) long exposure

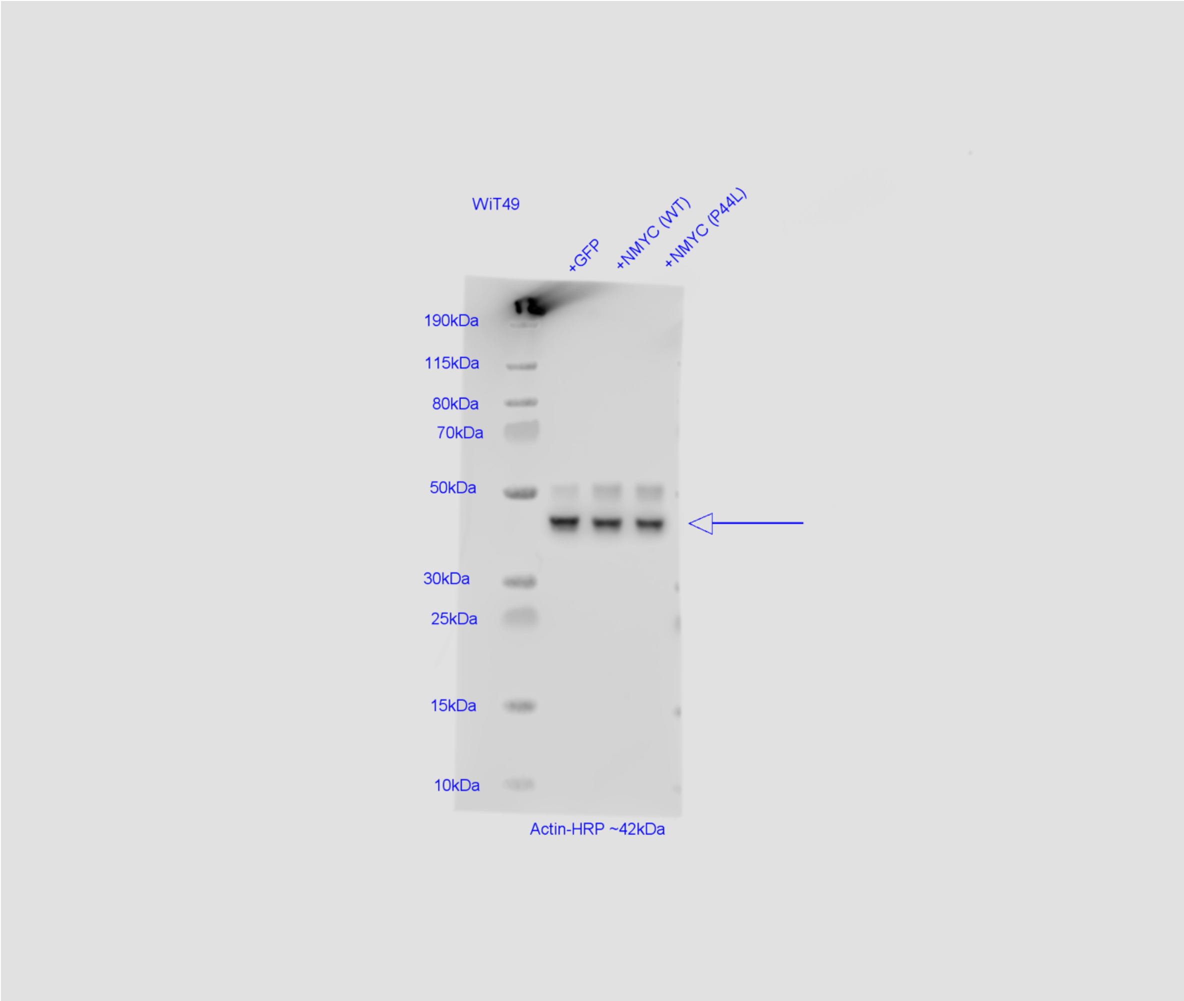

Figure 6c: Actin-HRP

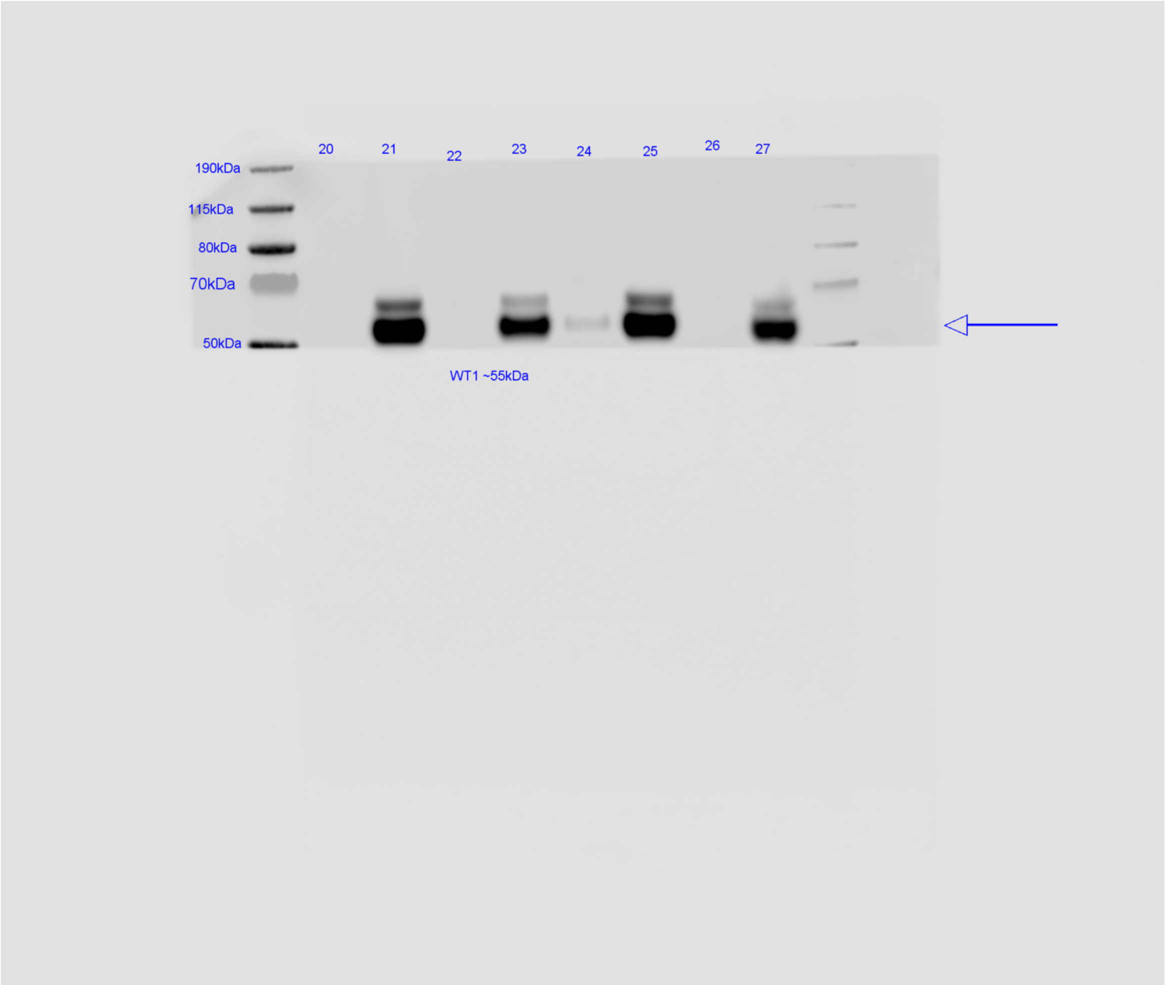

Supplementary Figure S3C: WT1 Xenografts KT 20-27

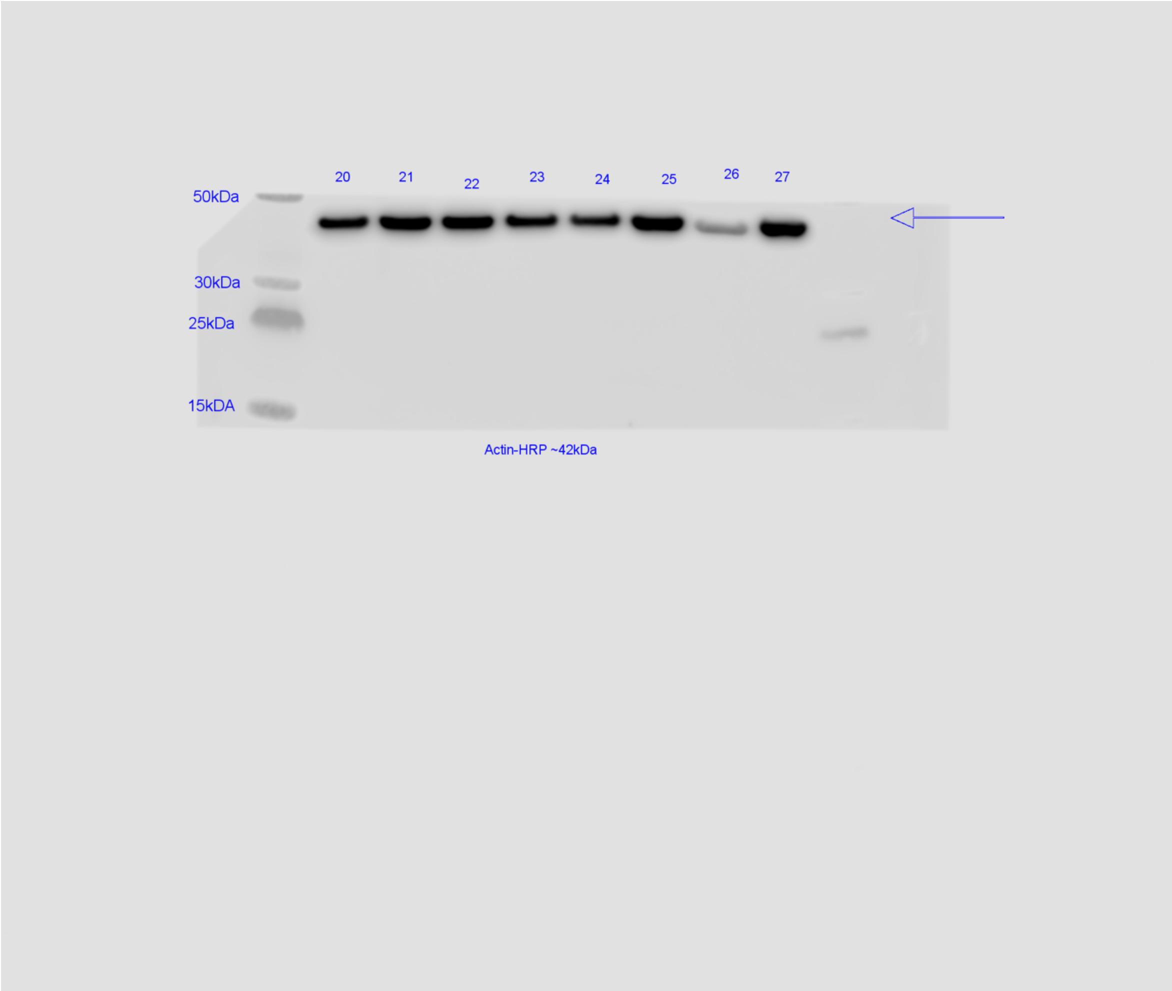

Supplementary Figure S3c: Actin-HRP xenografts KT20-27

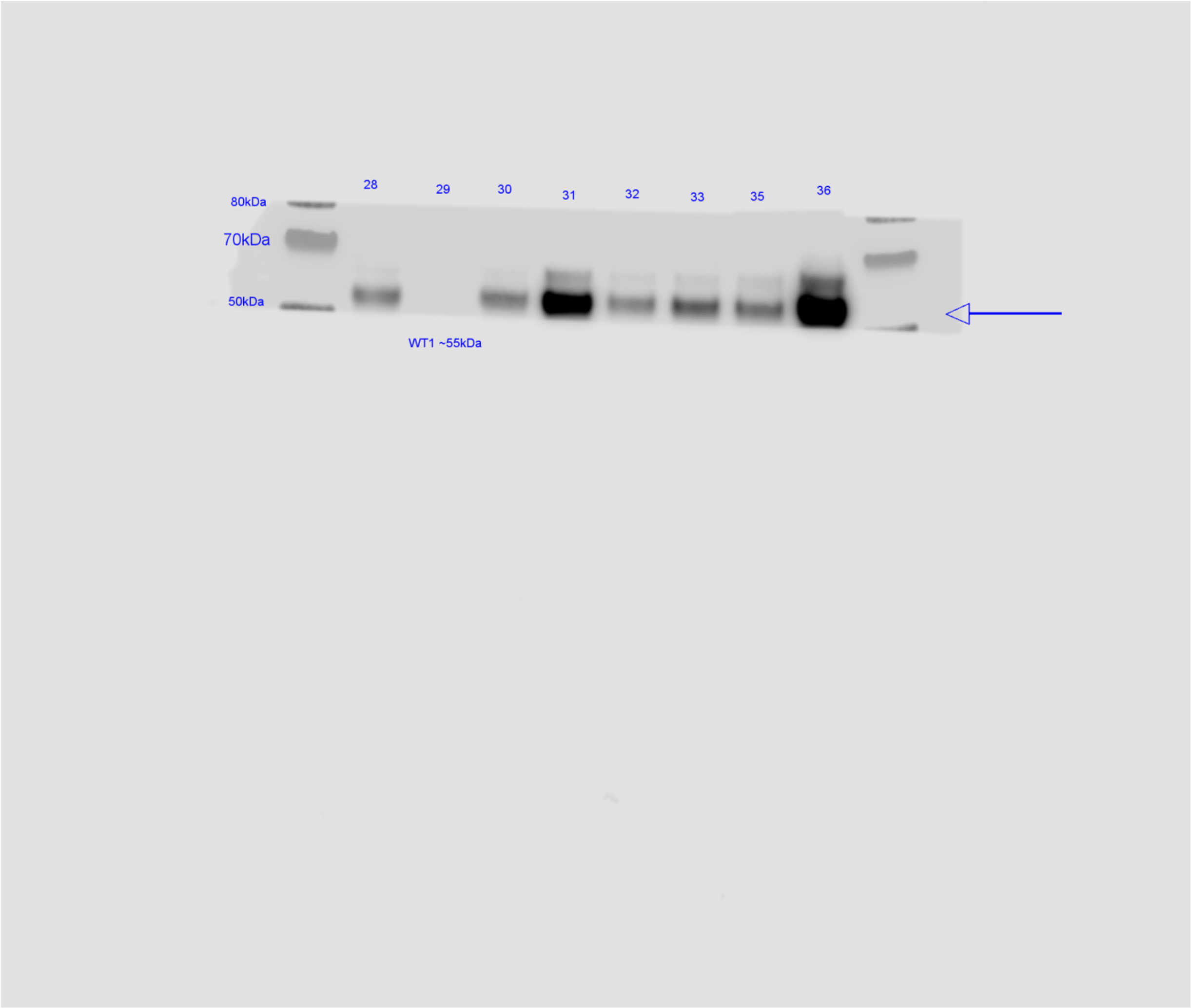

Supplementary Figure S3c: WT1 Xenografts KT28-36

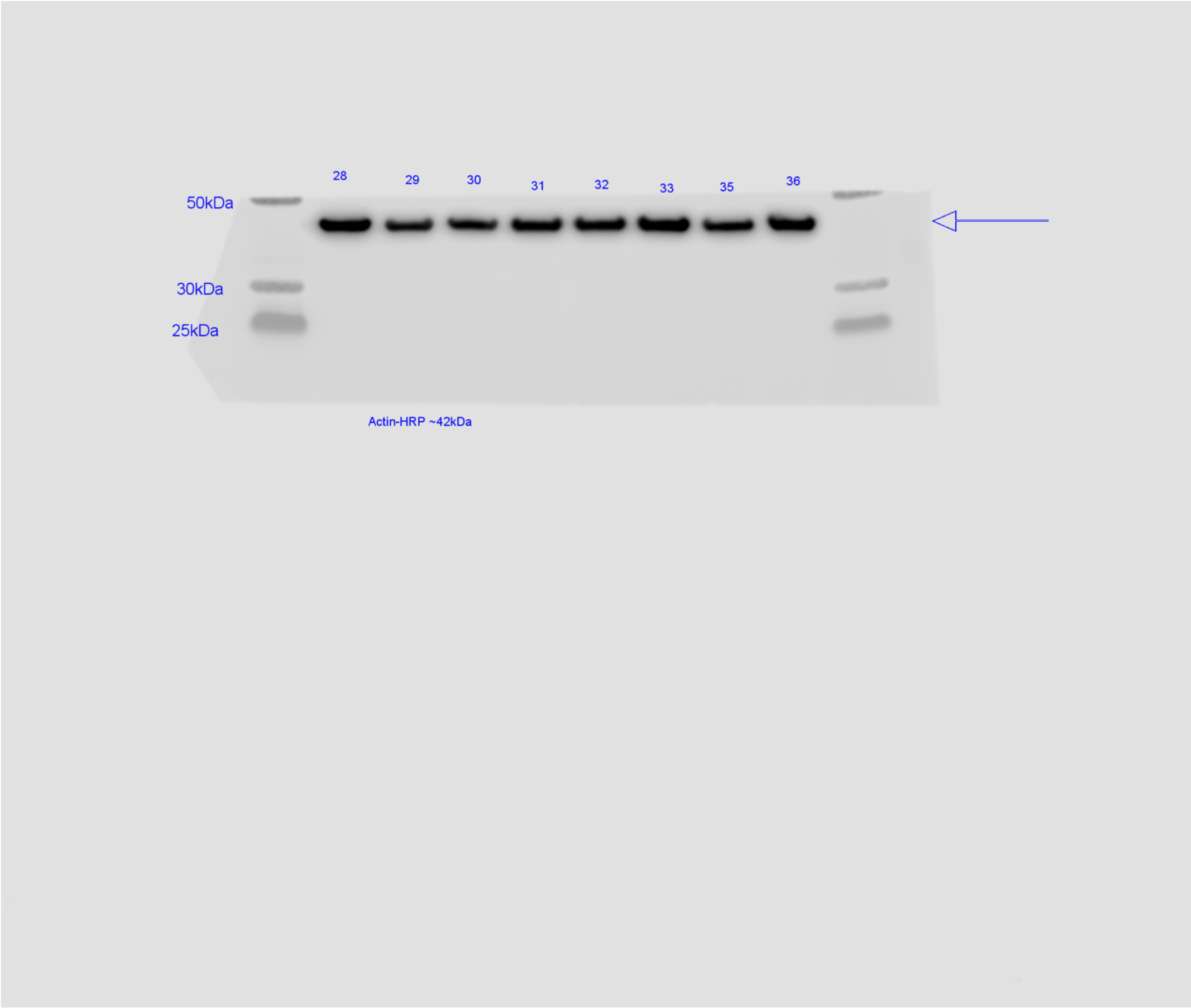

Supplementary Figure S3c: Actin-HRP Xenografts KT28-36

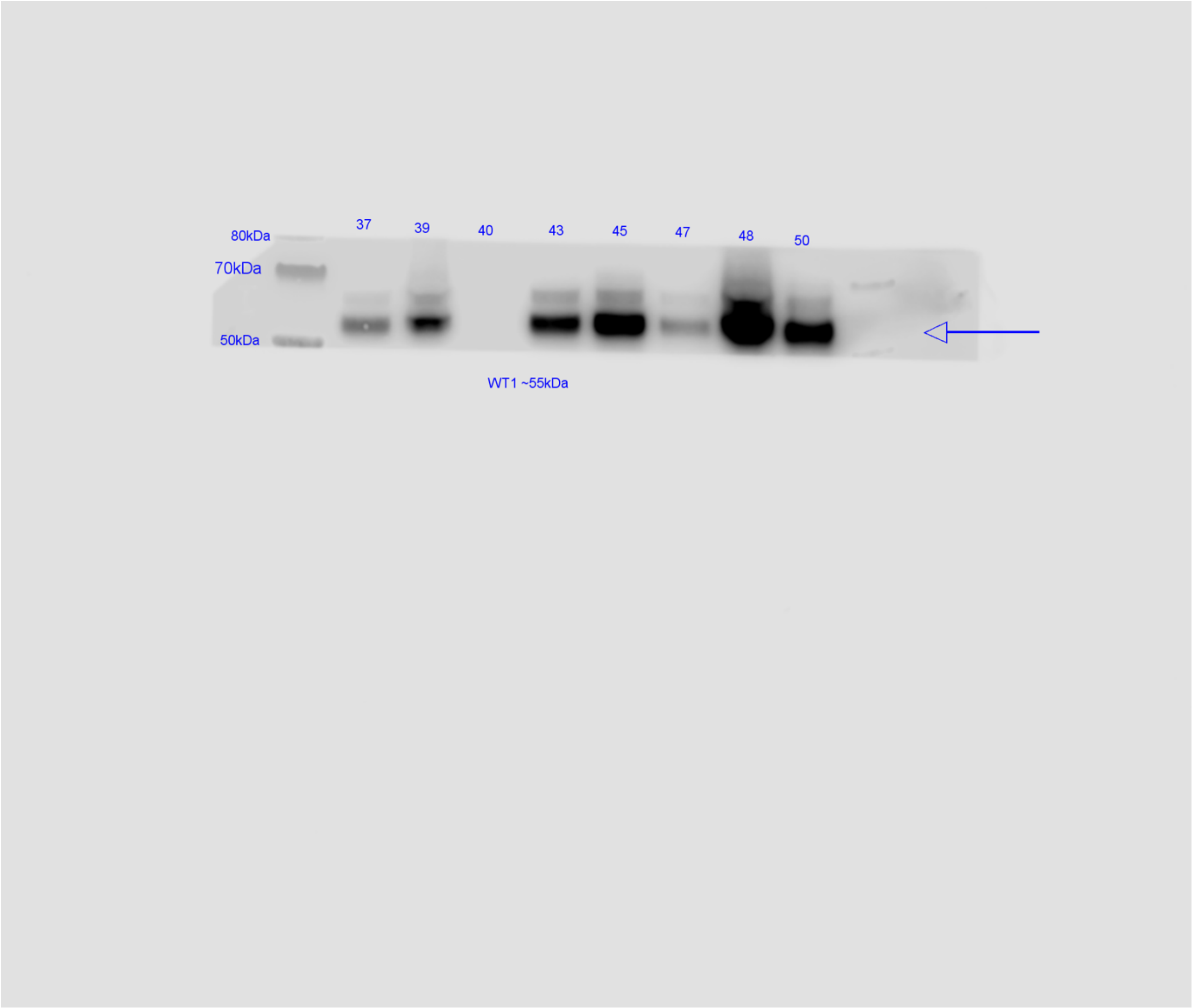

Supplementary Figure S3c: WT1 Xenografts KT37-50

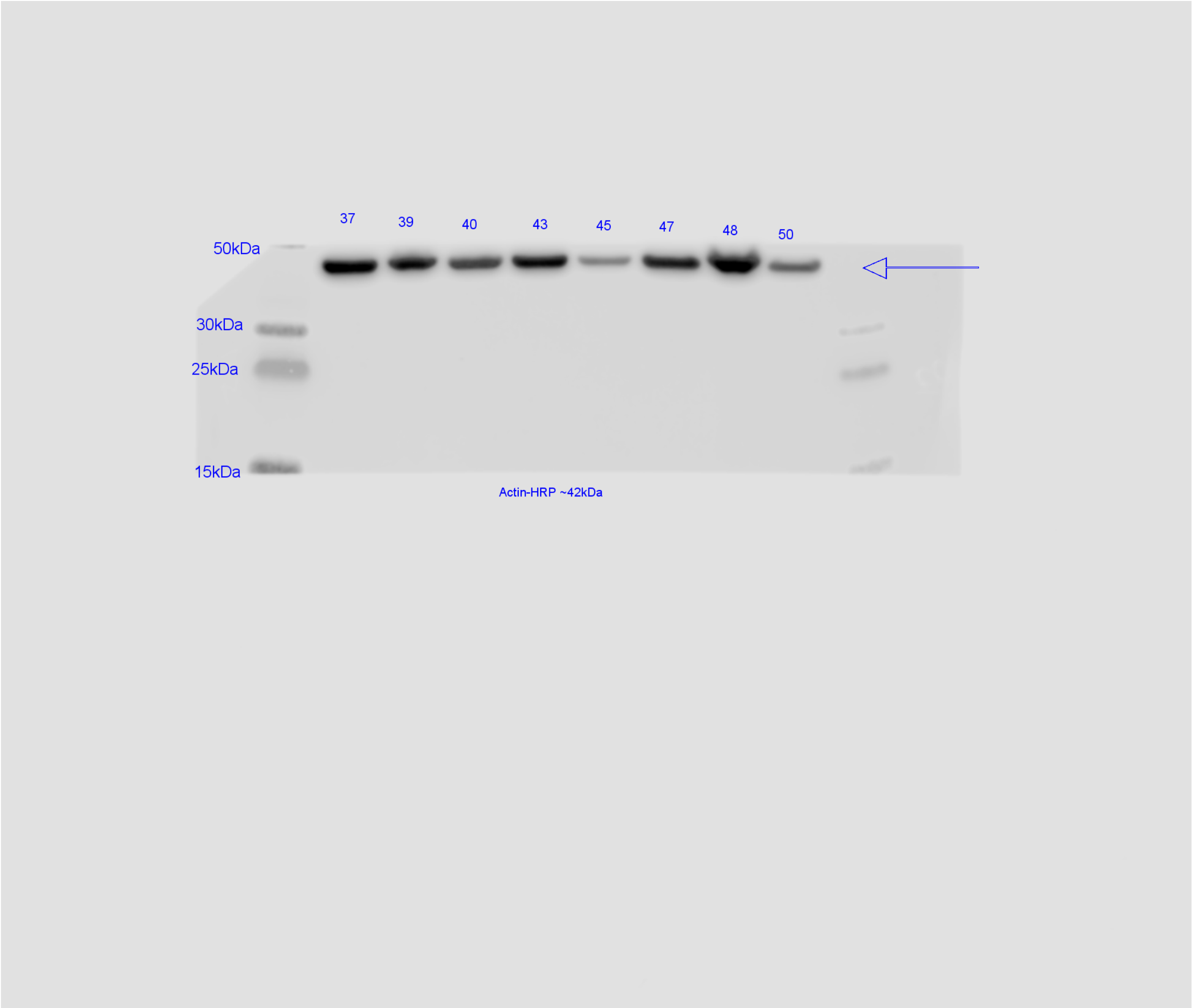

Supplementary Figure S3c: Actin-HRP Xenografts KT37-50

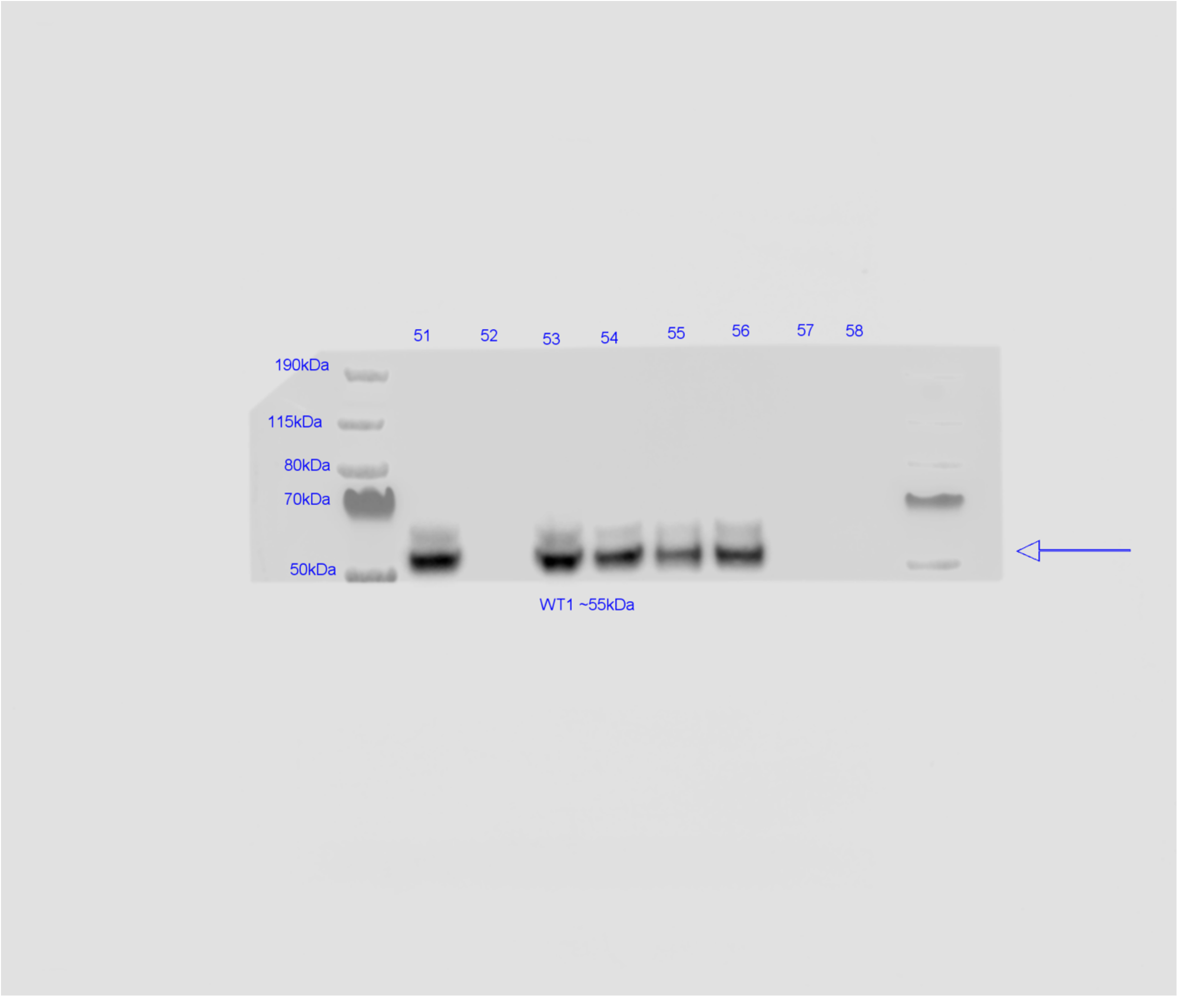

Supplementary Figure S3c: WT1 Xenografts KT51-58

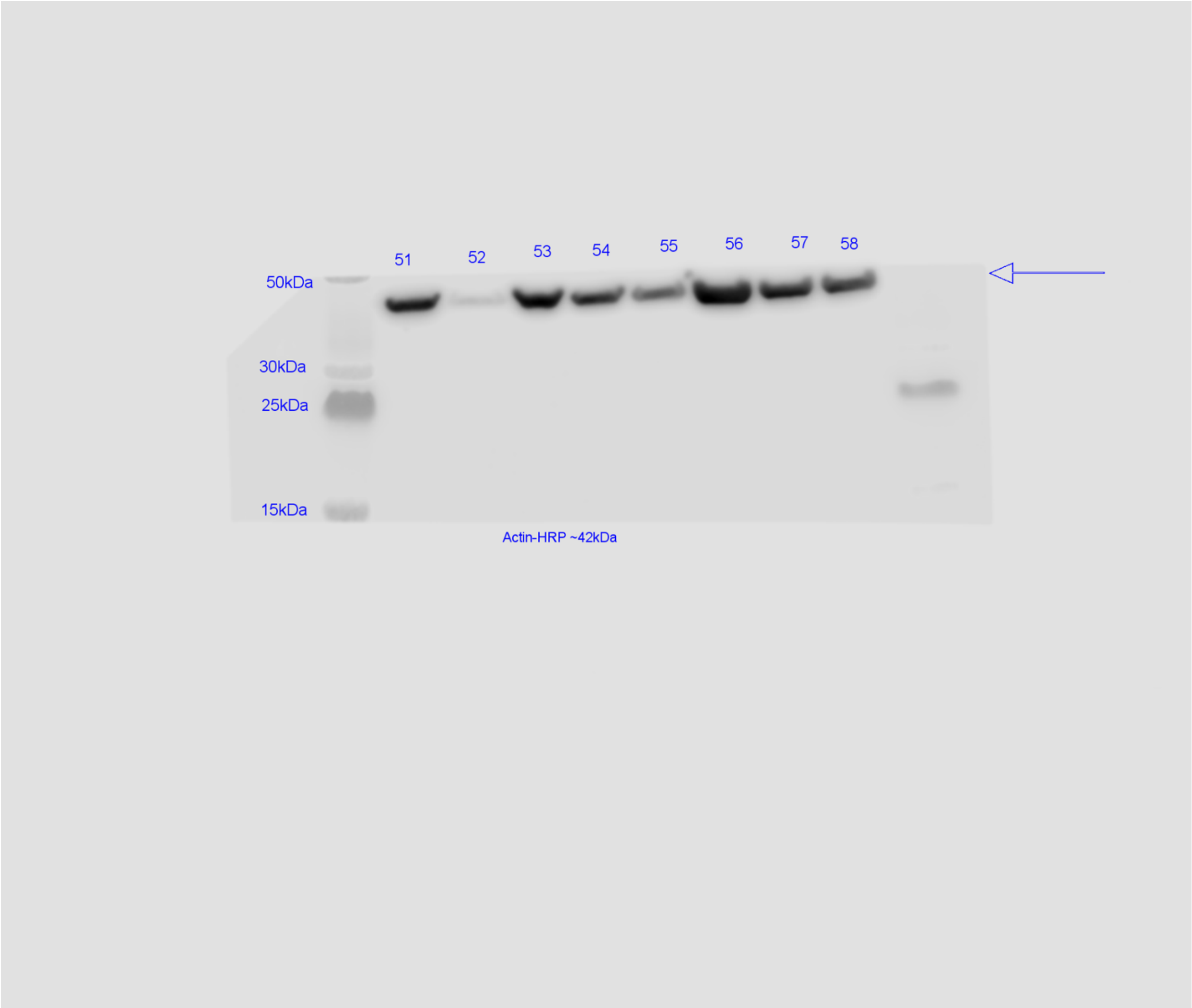

Supplementary Figure S3c: Actin-HRP Xenografts KT51-58

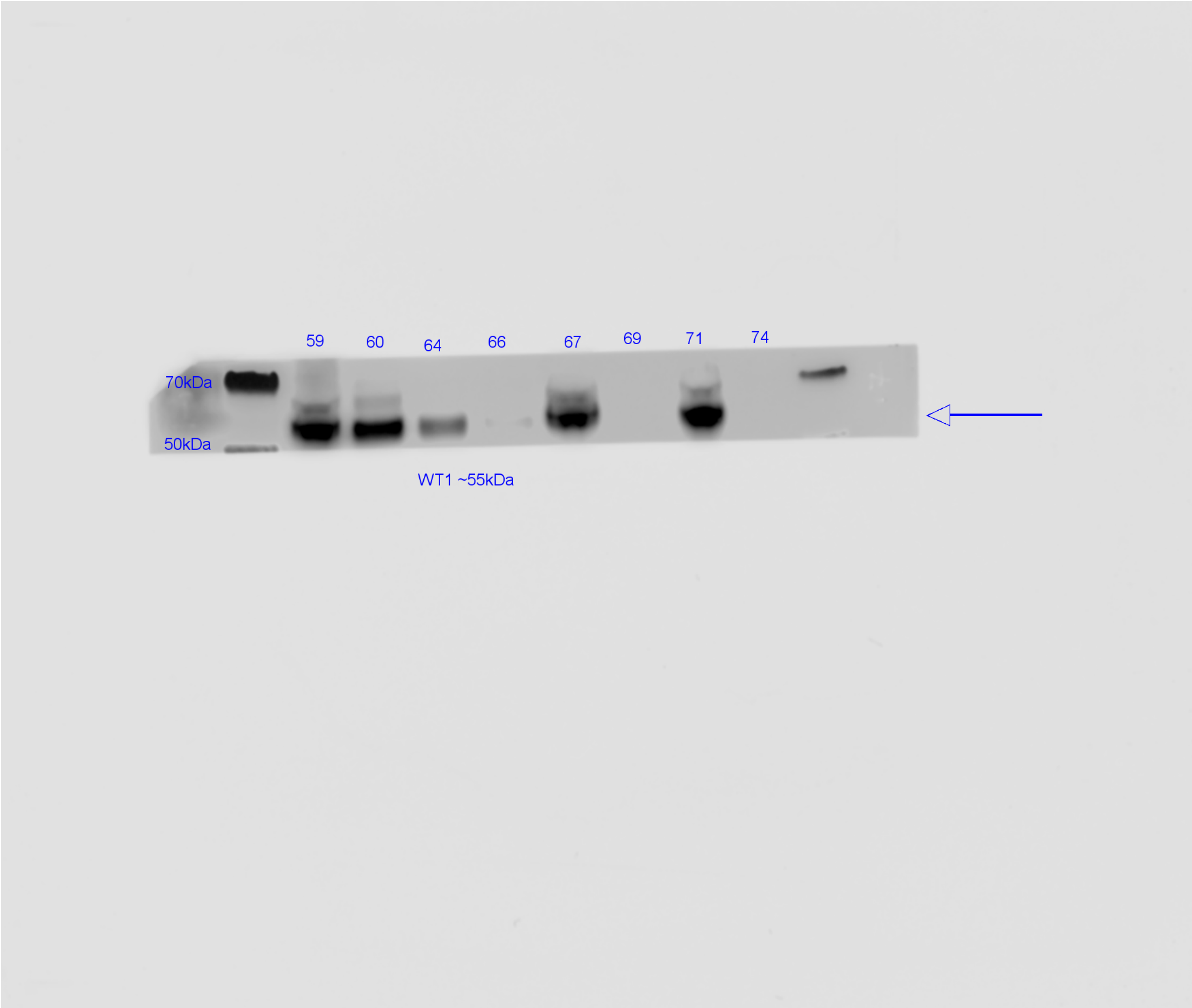

Supplementary Figure S3c: WT1 Xenografts KT59-74

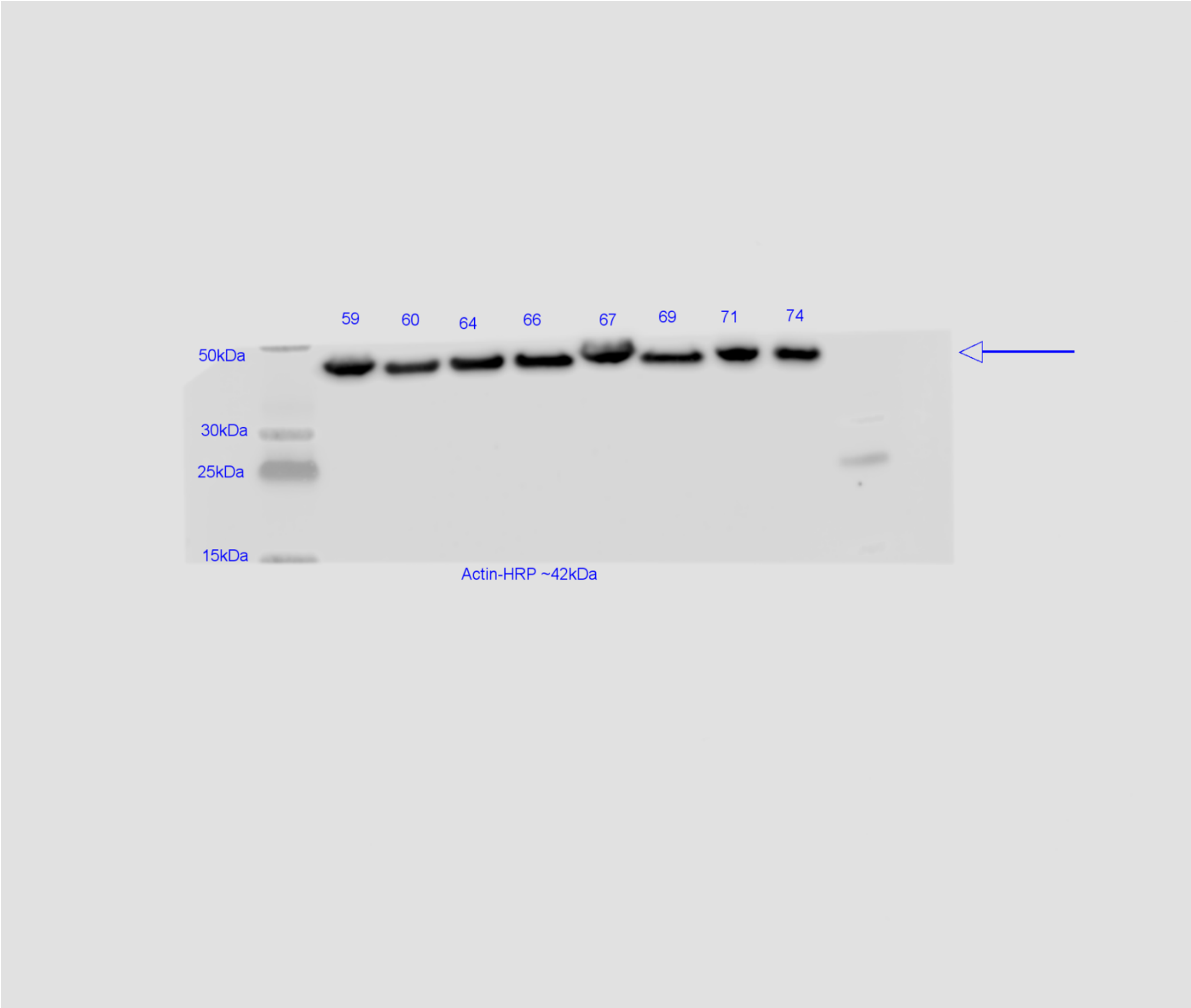

Supplementary Figure S3c: Actin-HRP Xenografts KT59-74

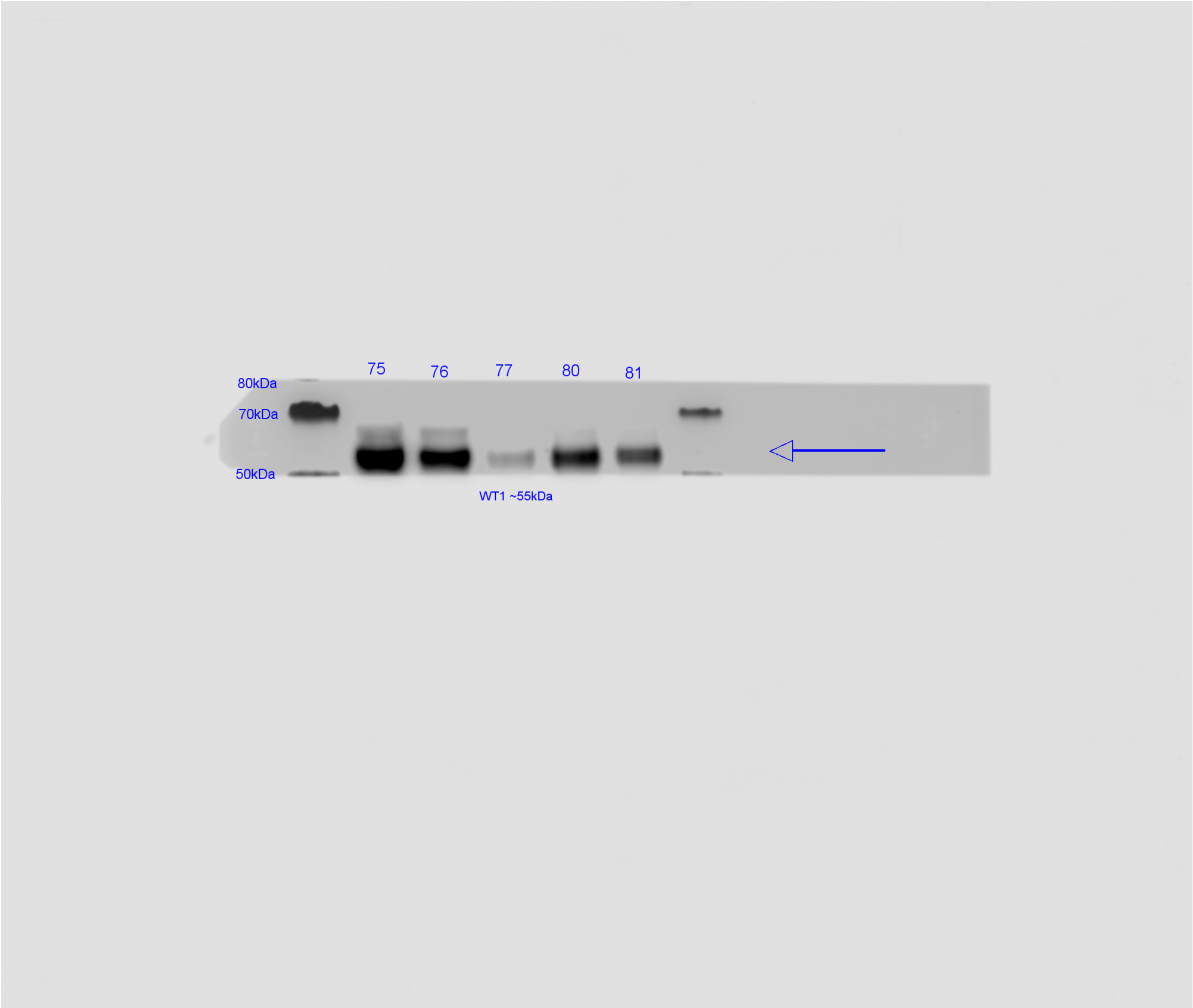

Supplementary Figure S3c: WT1 Xenografts KT75-81

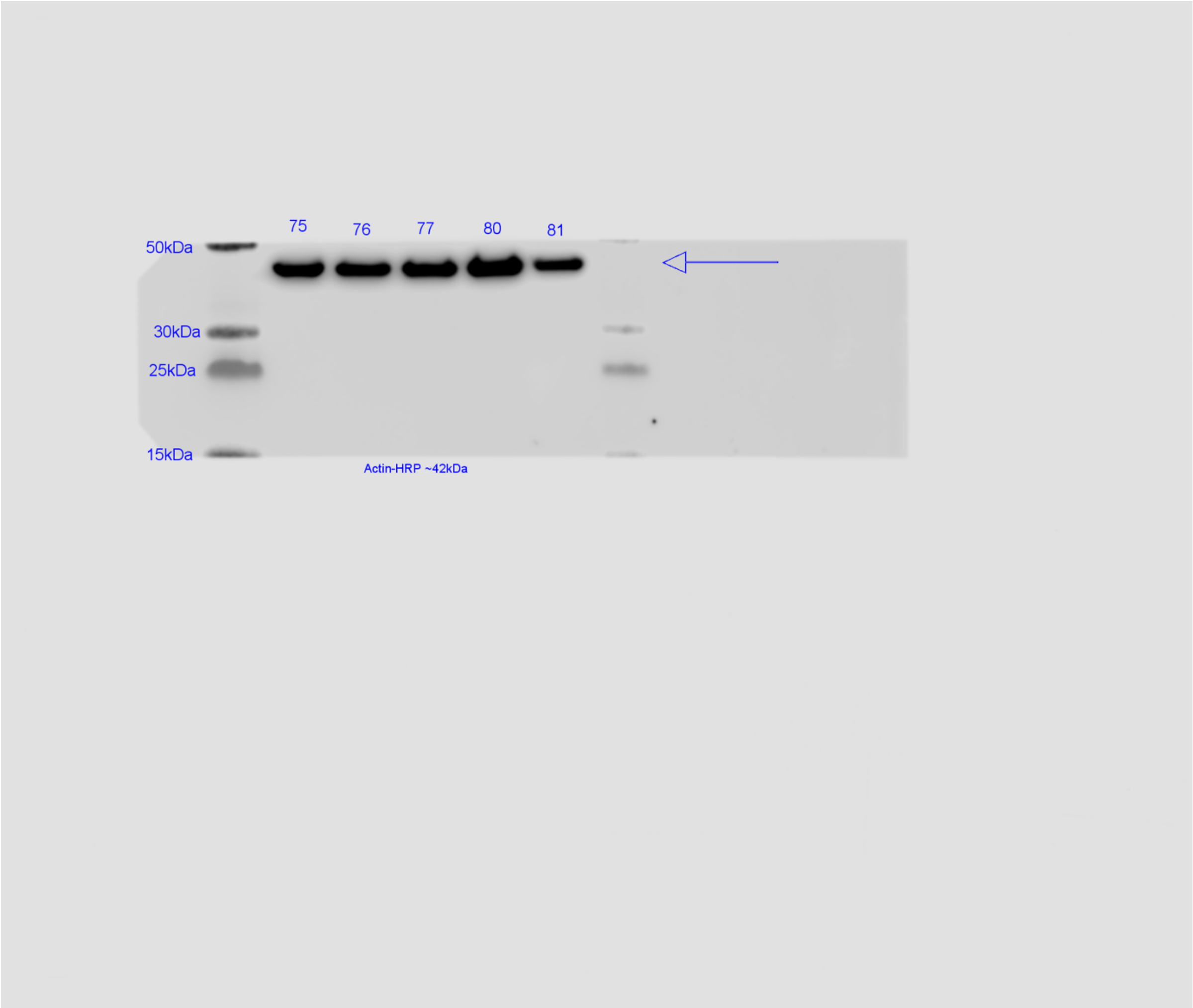

Supplementary Figure S3c: Actin-HRP Xenografts KT75-81

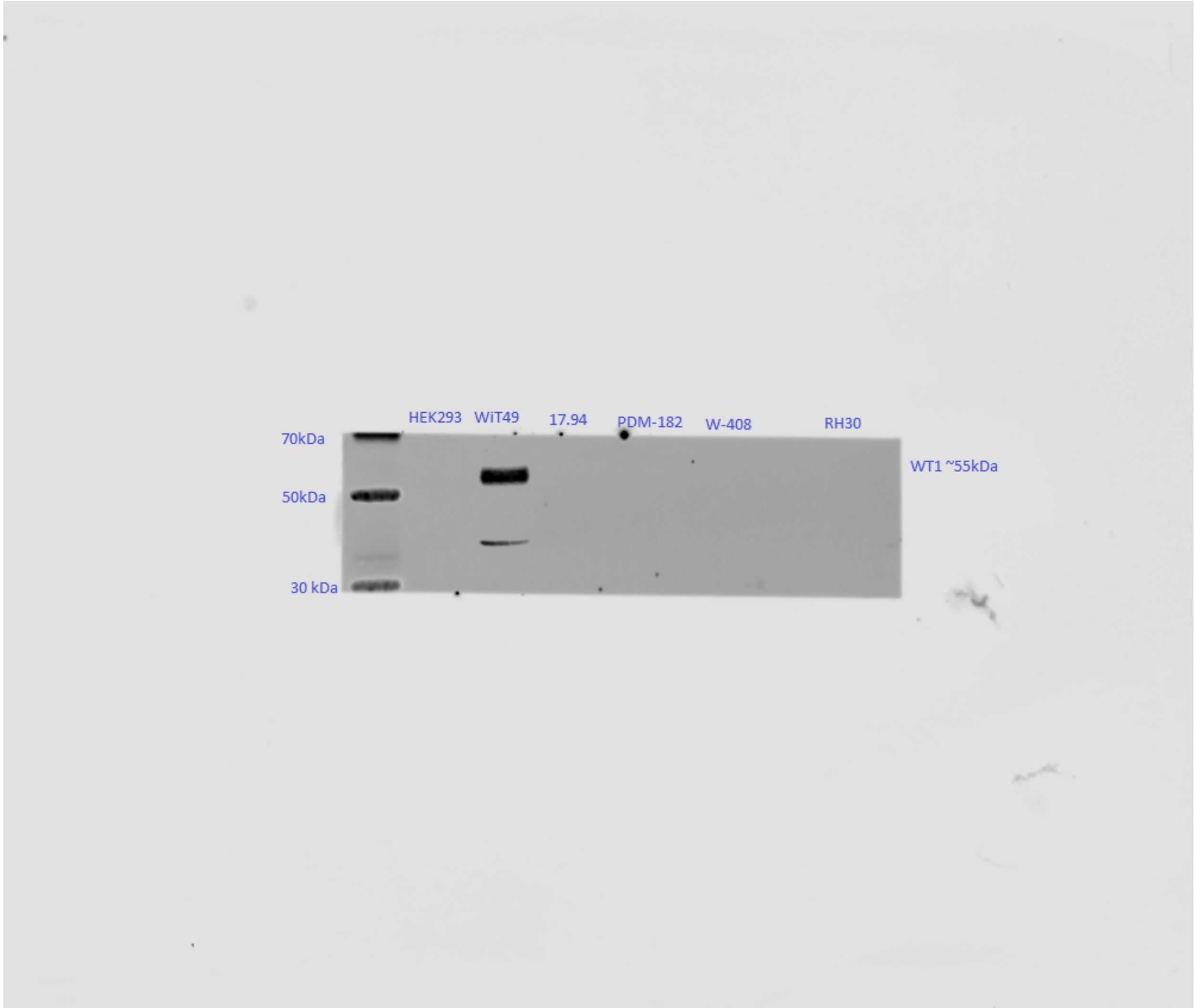

Supplementary Figure S5c: WT1

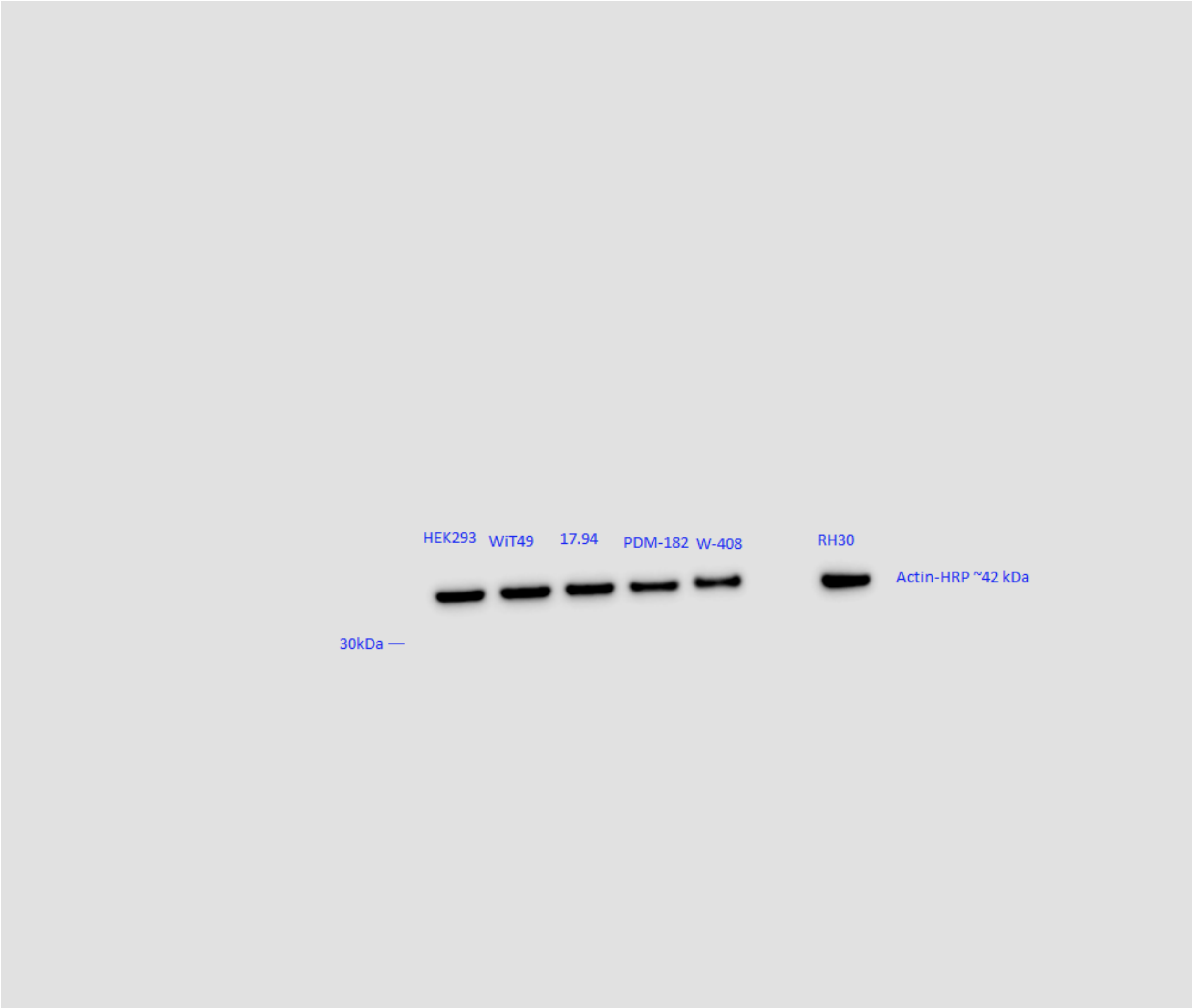

Supplementary Figure S5c: Actin-HRP

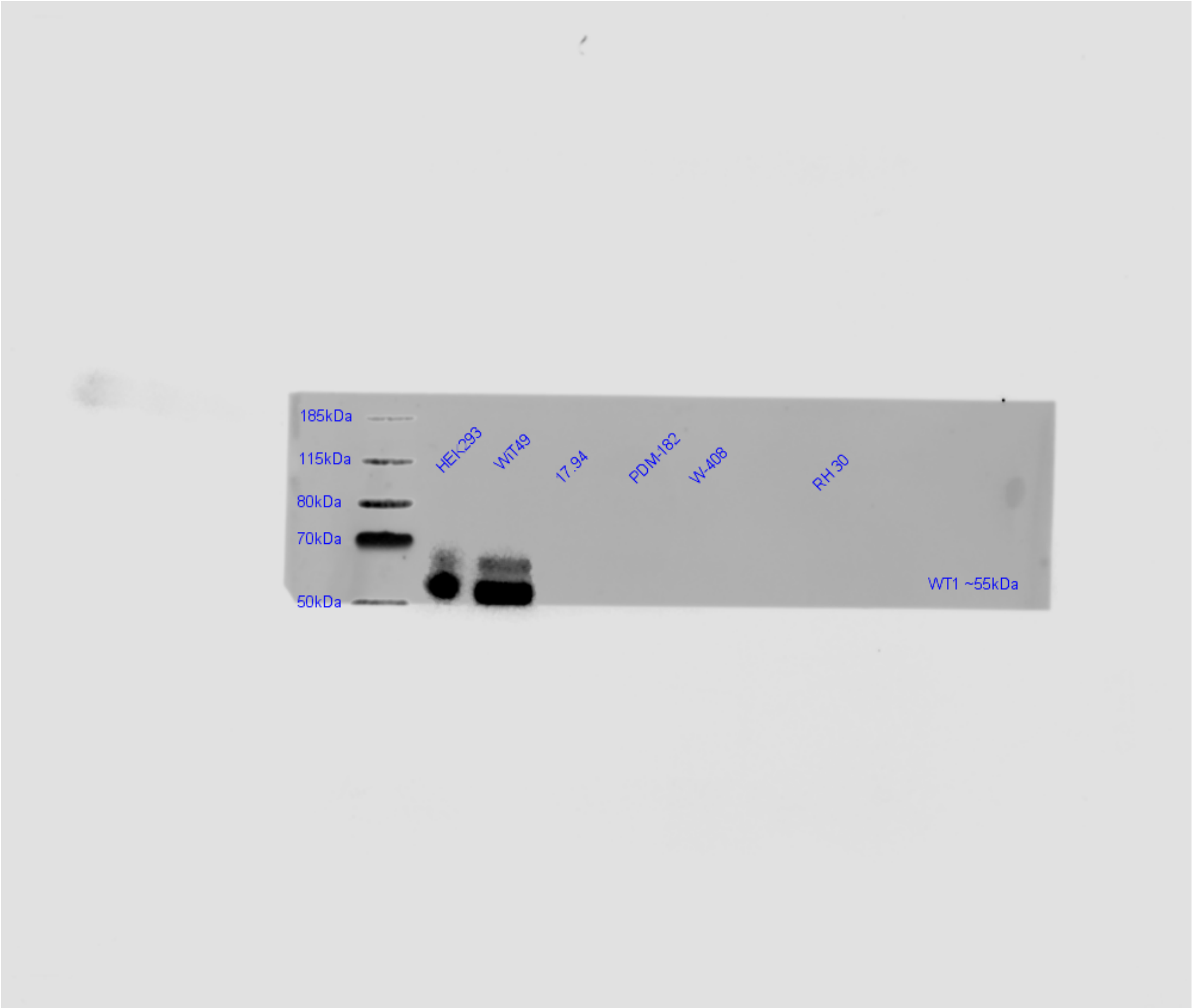

Supplementary Figure S5d: WT1

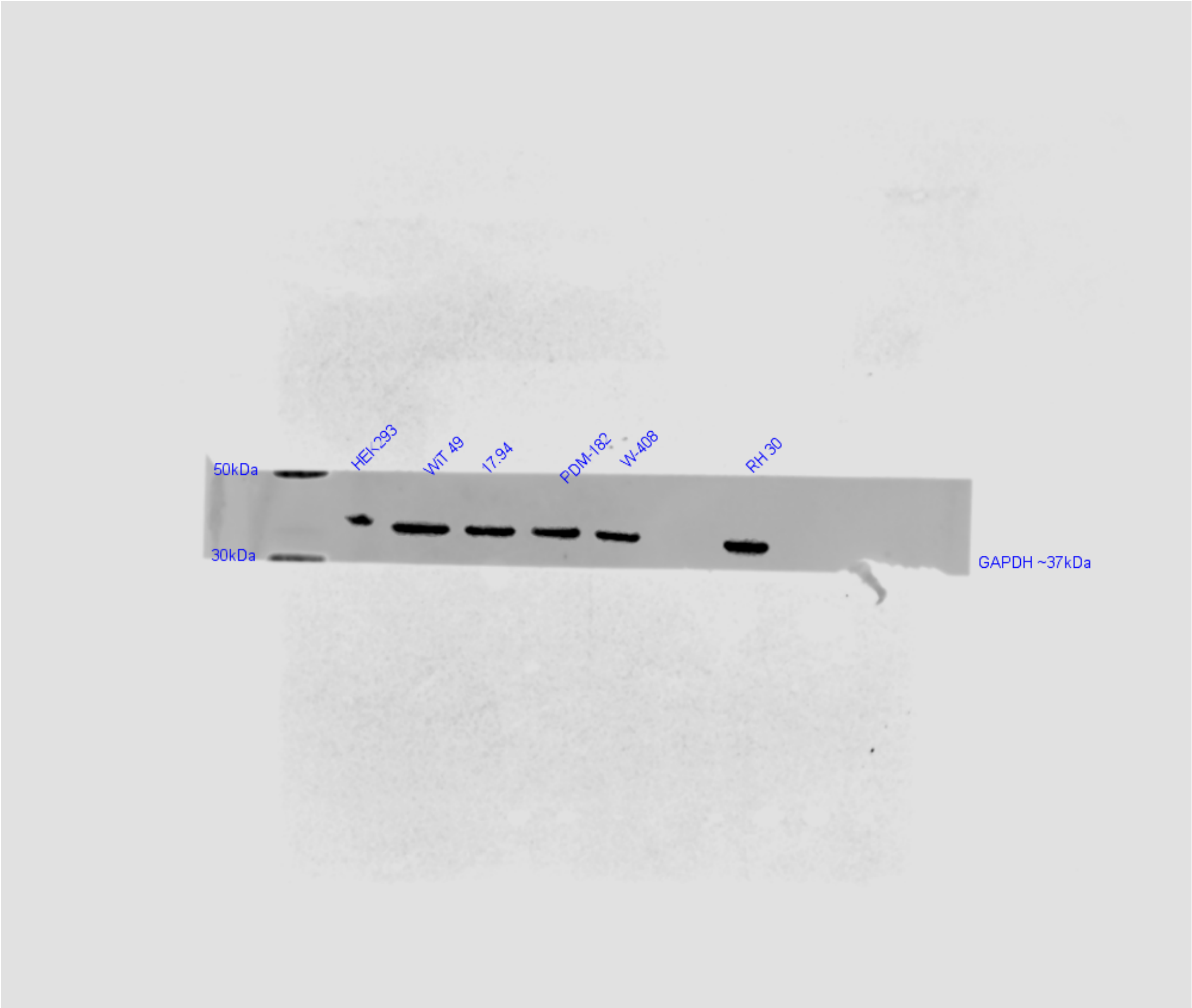

Supplementary Figure S5d: GAPDH

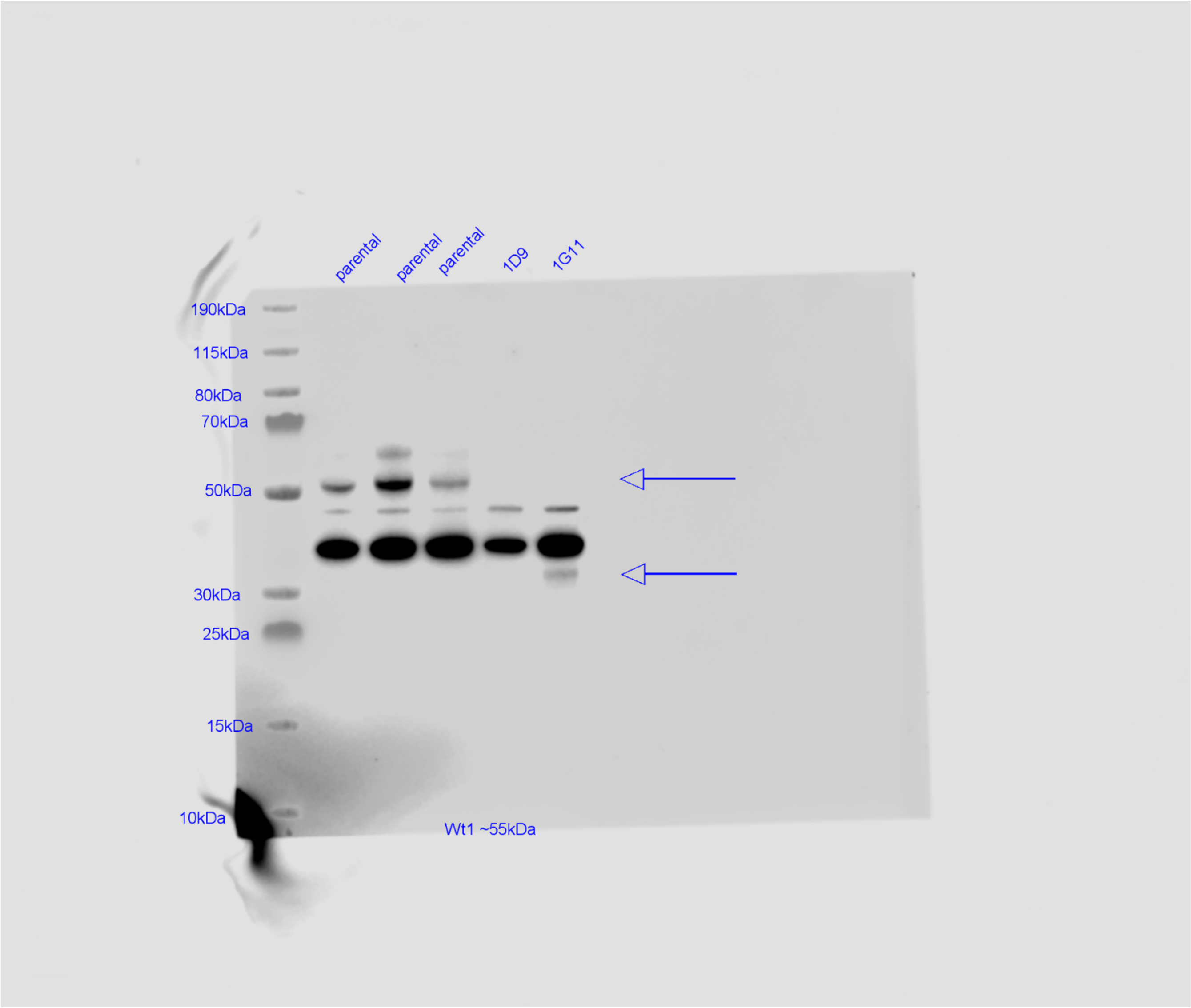

Supplementary Figure S7c: WT1

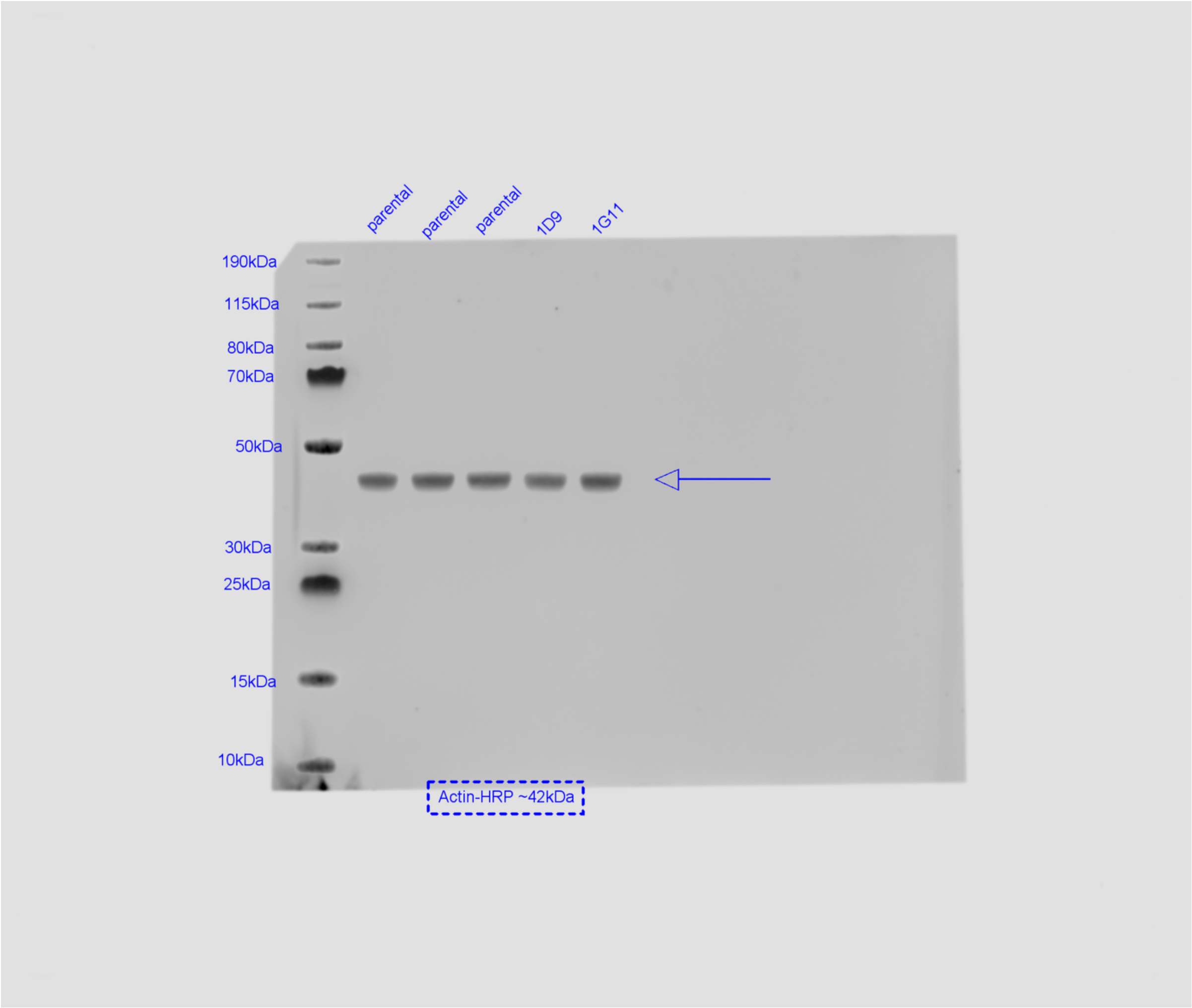

Supplementary Figure S7c: Actin-HRP

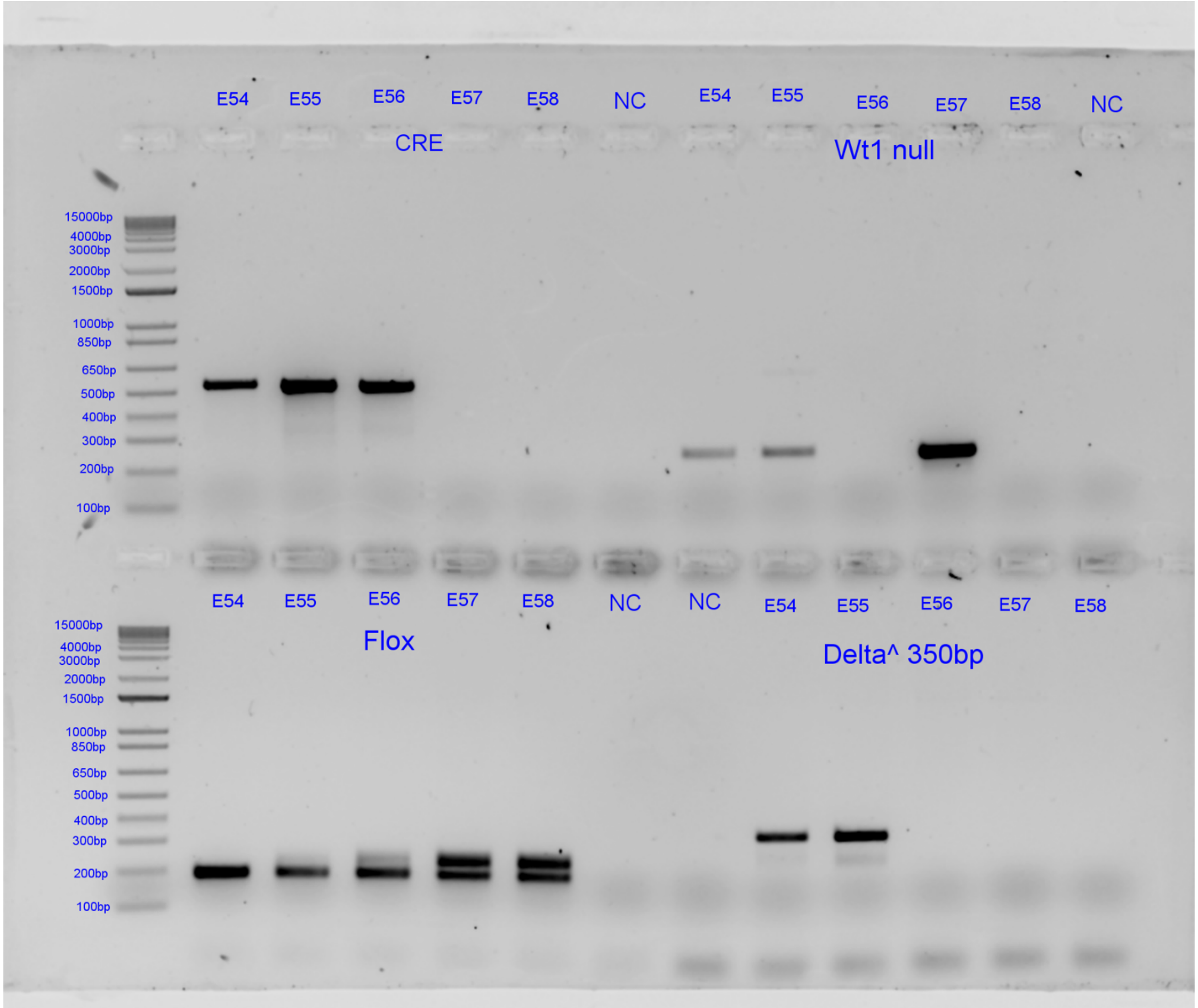

Supplementary Figure S8a: DNA PCR gel
