# Supplementary material for: TERT Expression in Wilms Tumor Is Regulated by Promoter Mutation or Hypermethylation, WT1, and N-MYC
Source: Cancers (Basel). 2022 Mar 25;14(7):1655. doi: 10.3390/cancers14071655 (PMC8996936; doi:10.3390/cancers14071655)
Supplement: Supplementary file 1 [file cancers-14-01655-s001.zip › TERT Supplementary Figures Revised 2-7-22.pdf]

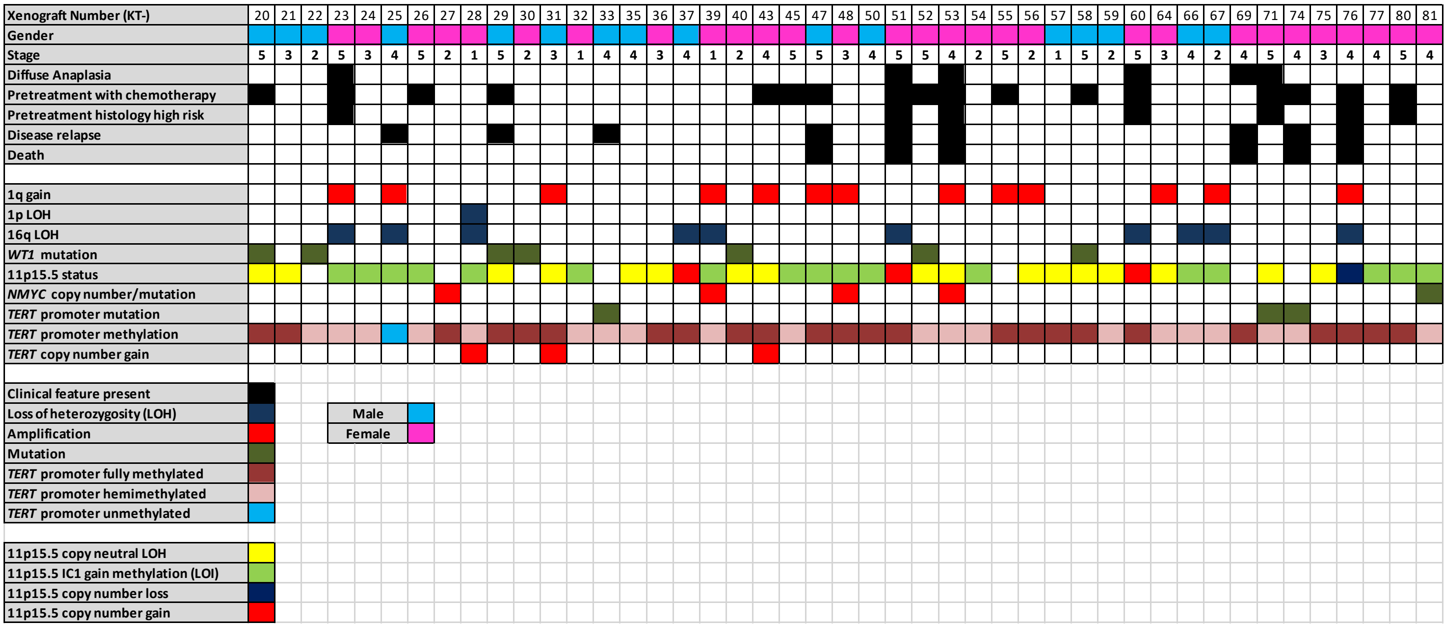

Supplementary Figure S1. Heatmap Summary of Molecular Data. Top panels show clinical data. Middle panels show molecular data reported in manuscript including *TERT* promoter mutations, *TERT* locus copy number gain, and *TERT* promoter methylation status. LOH – loss of heterozygosity. LOI – loss of imprinting (site specific gain of methylation at H19/ICR1).

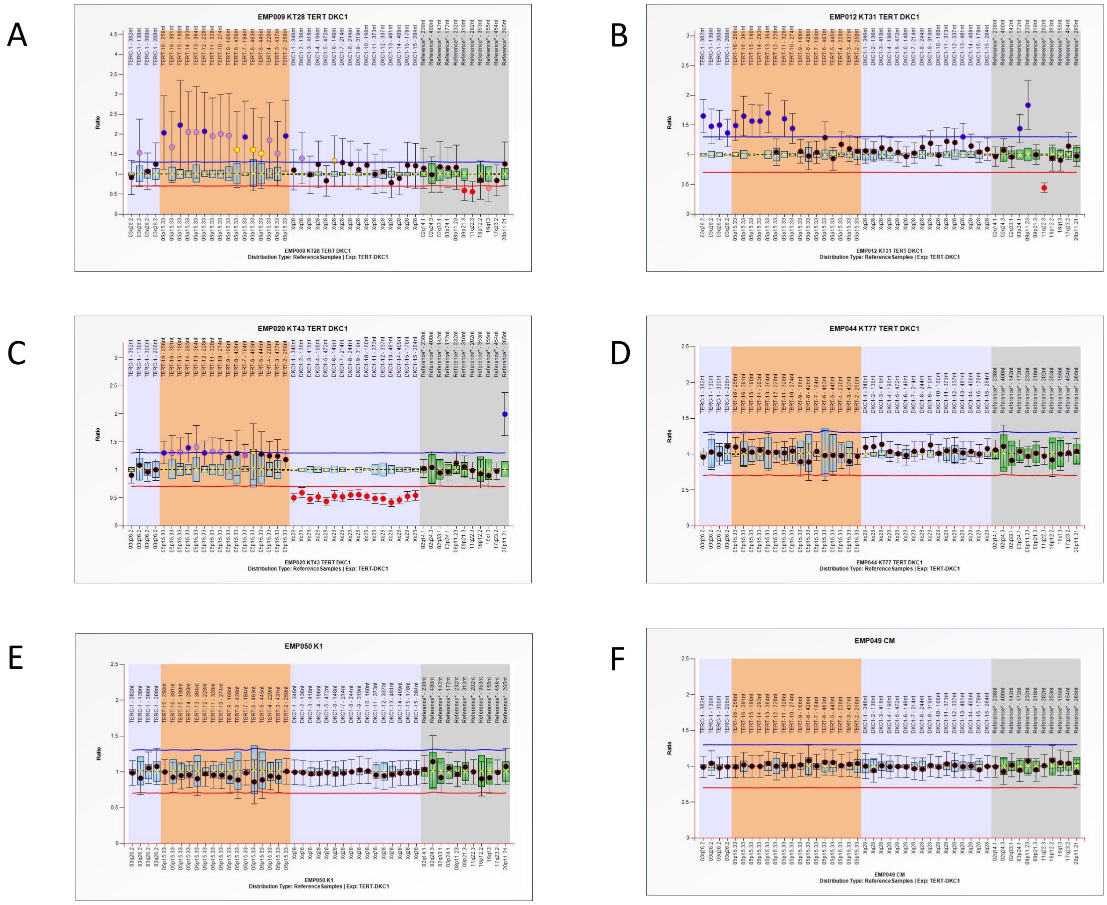

Supplementary Figure S 2. *TERT* Copy number gain in Wilms tumor patient-derived xenografts. Multiplex-ligation probe amplification (MLPA) plots demonstrate copy number gain of *TERT* (5p15.33, salmon color) in Wilms tumor patient-derived xenografts (A) KT-28, (B) KT-31, and (C) KT-43. For contrast a diploid copy number profile is shown for (D) KT-77, (E) a normal kidney specimen, and (F) commercially-available control normal male DNA.

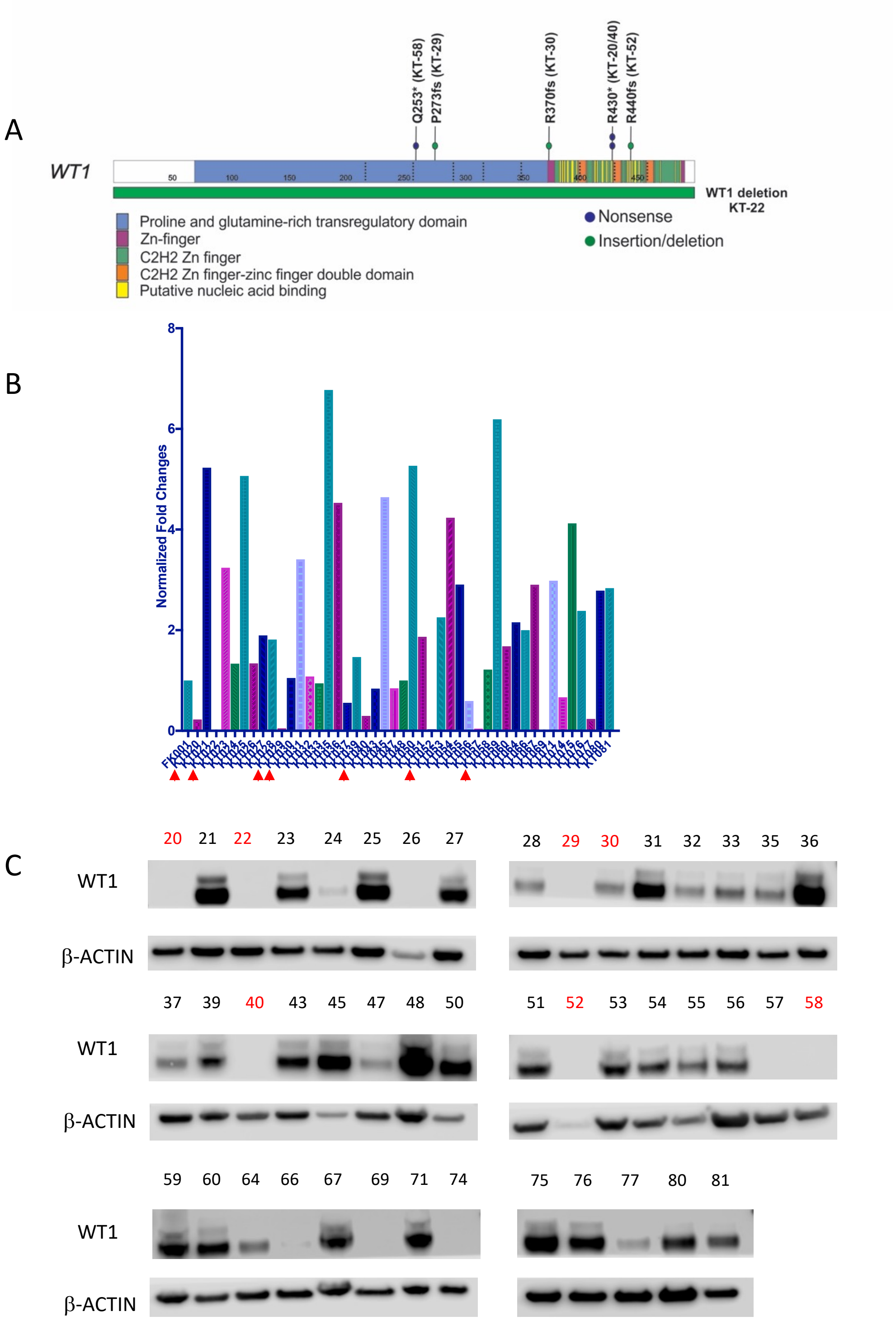

Supplementary Figure S3. *WT1* mutations and expression of WT1 in Wilms tumor patient-derived xenografts. (A) Seven xenograft specimens (KT-20, 22, 29, 30, 40, 52, and 58) were found to have biallelic inactivating mutations in *WT1* by Sanger sequencing. (B) The relative gene expression of *WT1* as determined by qRT-PCR normalized to fetal kidney (FK001- first sample on x-axis) expression is shown. Red arrows indicate *WT1*-mutant specimens. (C) Protein expression of WT1 is shown with xenografts shown in red font indicating specimens determined to have biallelic inactivating mutations in *WT1*.

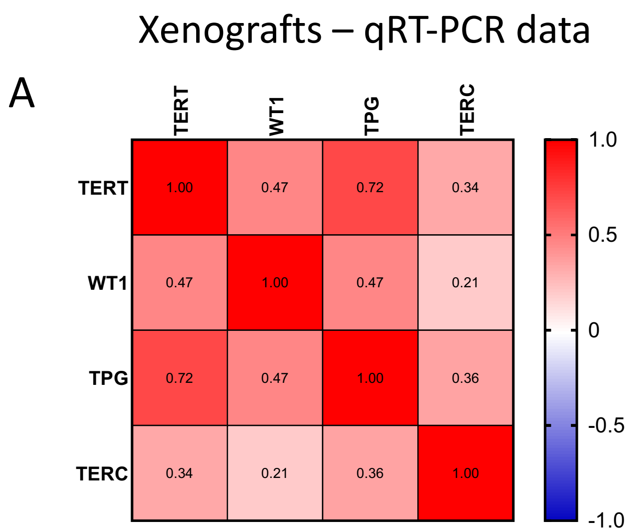

P-values

	TERT	WT1	TPG	TERC
TERT		0.001	3.3x10 3-8	0.023
WT1	0.001		0.001	0.176
TPG	3.3x10 3-8	0.001		0.016
TERC	0.023	0.176	0.016	

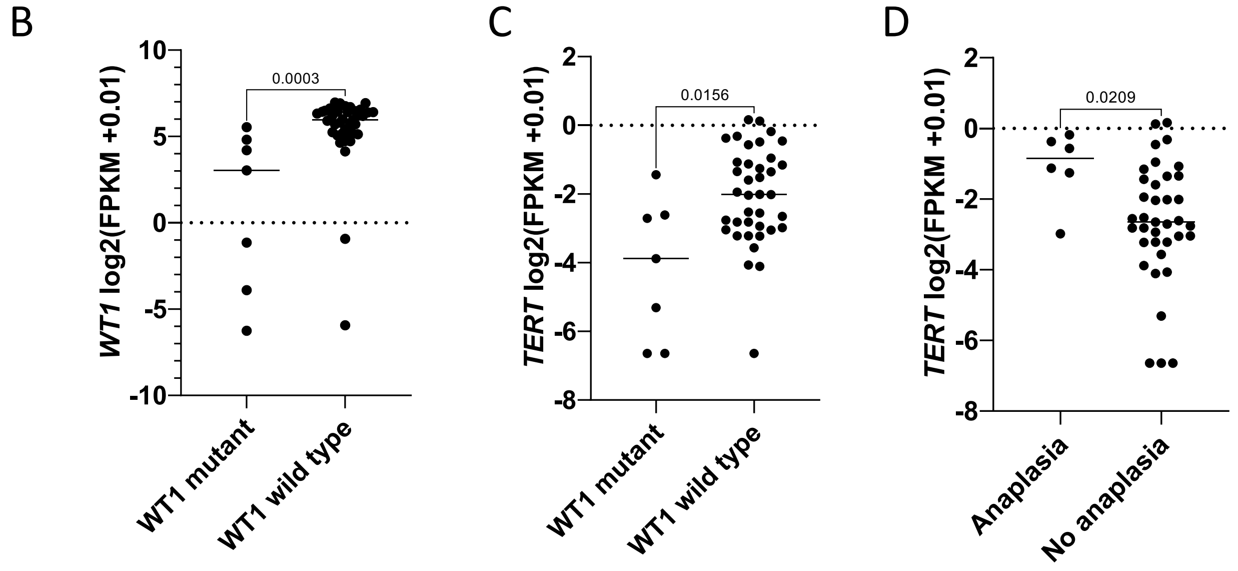

Supplementary Figure S4. (A) qRT-PCR demonstrates statistically significant positive correlations between *TERT* expression and telomerase activity (TPG) and *WT1* expression and telomerase activity (TPG). Correlations are Spearman r values with associated p-values in right panel. (B) The statistically significant reduction in WT1 expression among xenograft samples with inactivating *WT1* mutations was confirmed by RNA-seq (Mann-Whitney p value = 0.0003). (C) Xenograft specimens with inactivating mutations in WT1 were found to have lower *TERT* expression by RNA-seq (p=0.0156). (D) Xenograft samples with diffuse anaplasia were found to have significantly higher expression of *TERT* than favorable histology xenografts by RNA-seq (p=0.0209).

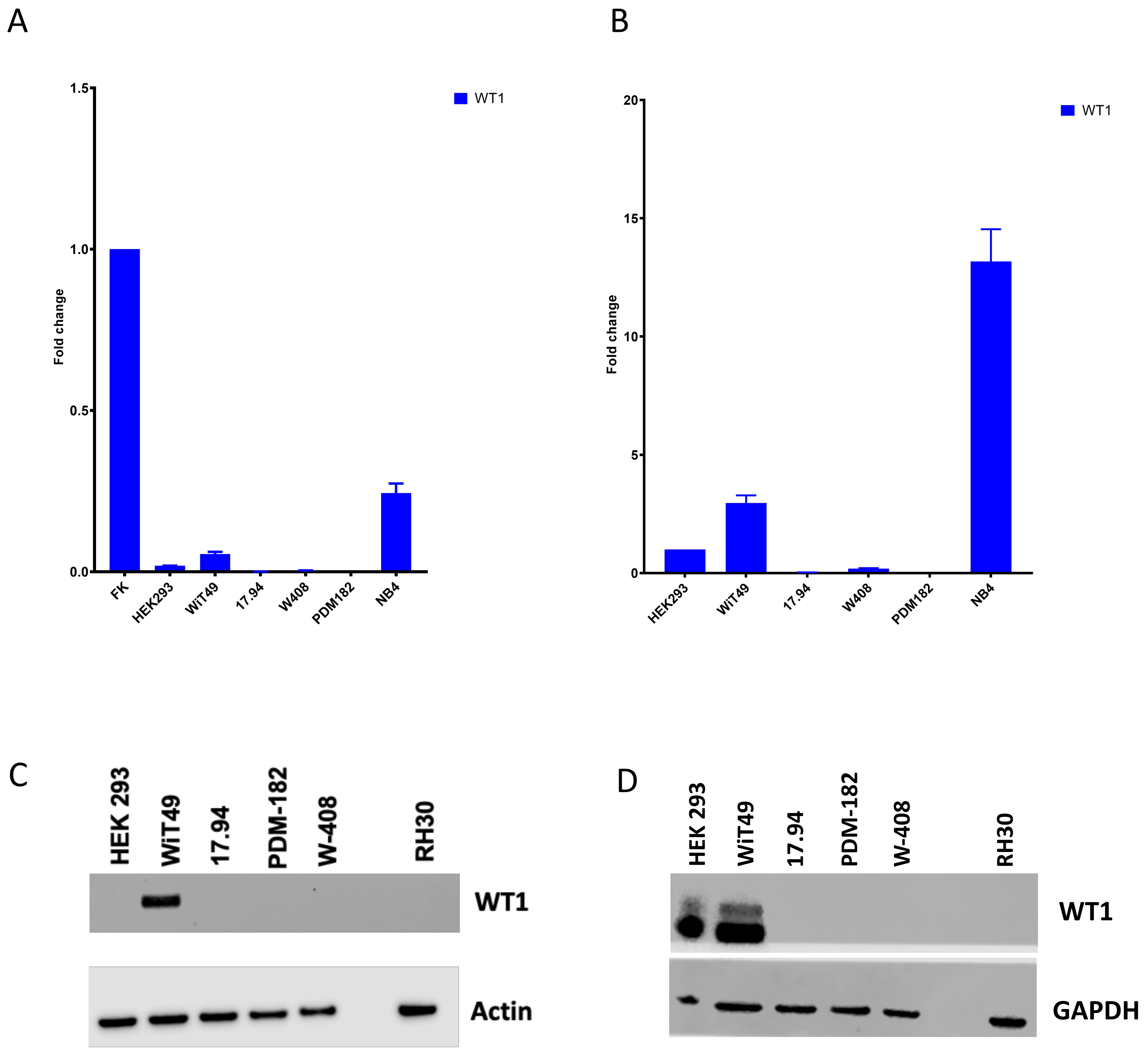

Supplementary Figure S5. Expression of WT1 in *in vitro* cell lines. (A) The relative gene expression of *WT1* determined by qRT-PCR and normalized to fetal kidney is shown. WT1 was detected in the transformed human embryonic kidney cell line HEK293, the anaplastic Wilms tumor cell line WiT49, and the human acute promyelocytic cell line NB4, but not the Wilms tumor cell lines 17.94, COGW408, or PDM182. (B) The relative expression of WT1 in these cell lines is shown using HEK293 as a reference. (C) Western blot demonstrates protein detection of WT1 in the WiT49 cell line. (D) In a different experiment and with longer exposure, western blot detection of WT1 is shown in HEK293 and WiT49 cells.

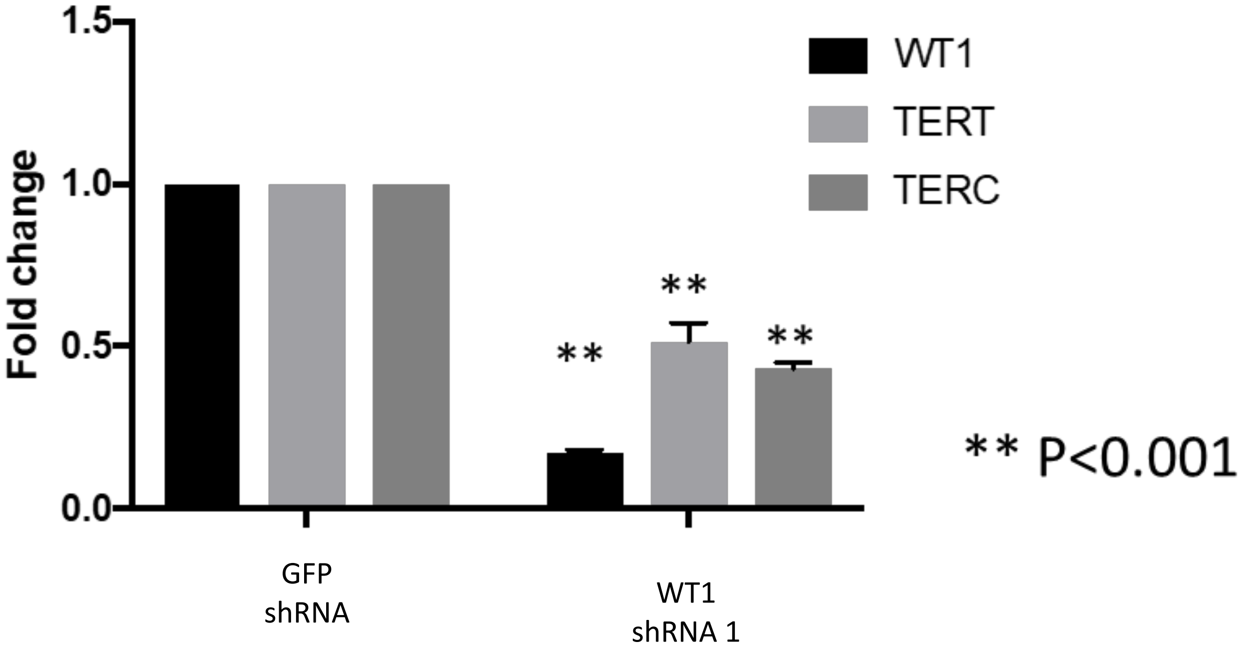

Supplementary Figure S6. WT1 knockdown in the acute promyelocytic cell line NB4 is associated with reduction in *TERT* transcription by qRT-PCR.

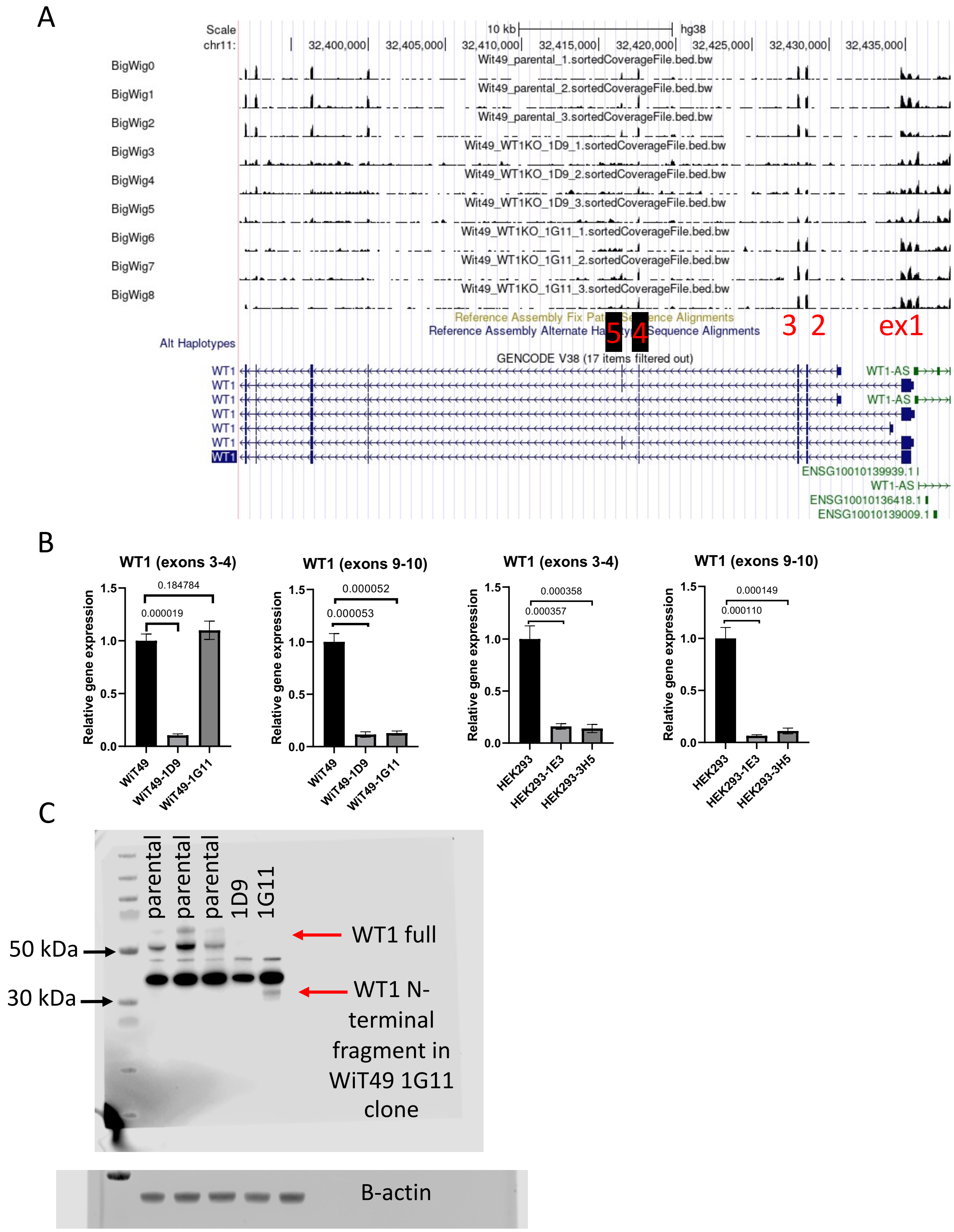

Supplementary Figure S7. Preservation of an N-terminal fragment in the WiT49 WT1 knockout (KO) clone WiT49-1G11 is associated with higher *TERT* expression. (A) Analysis of the RNA-seq reads using the UCSC Genome Browser shows preserved expression of WT1 exons 1,2,3,4, and possibly 5 in WT1-KO WiT49-1G11 cells, but not WiT49-1D9 cells. (B) This finding is confirmed using qRT-PCR with taqman probes targeted to the exon 3-4 junction of WT1 versus the exon 9-10 junction. The WT1-1G11 clone demonstrates persistent detection of the exon 3-4 fragment, but not the exon 9-10 fragment when compared to parental cells. In contrast, the other WT1-KO clones demonstrate consistently decreased detection of WT1 across all exons. (C) Western blot shows a WT1 protein fragment consistent with the predicted molecular weight of the N-terminal fragment in WiT49-1G11 cells.

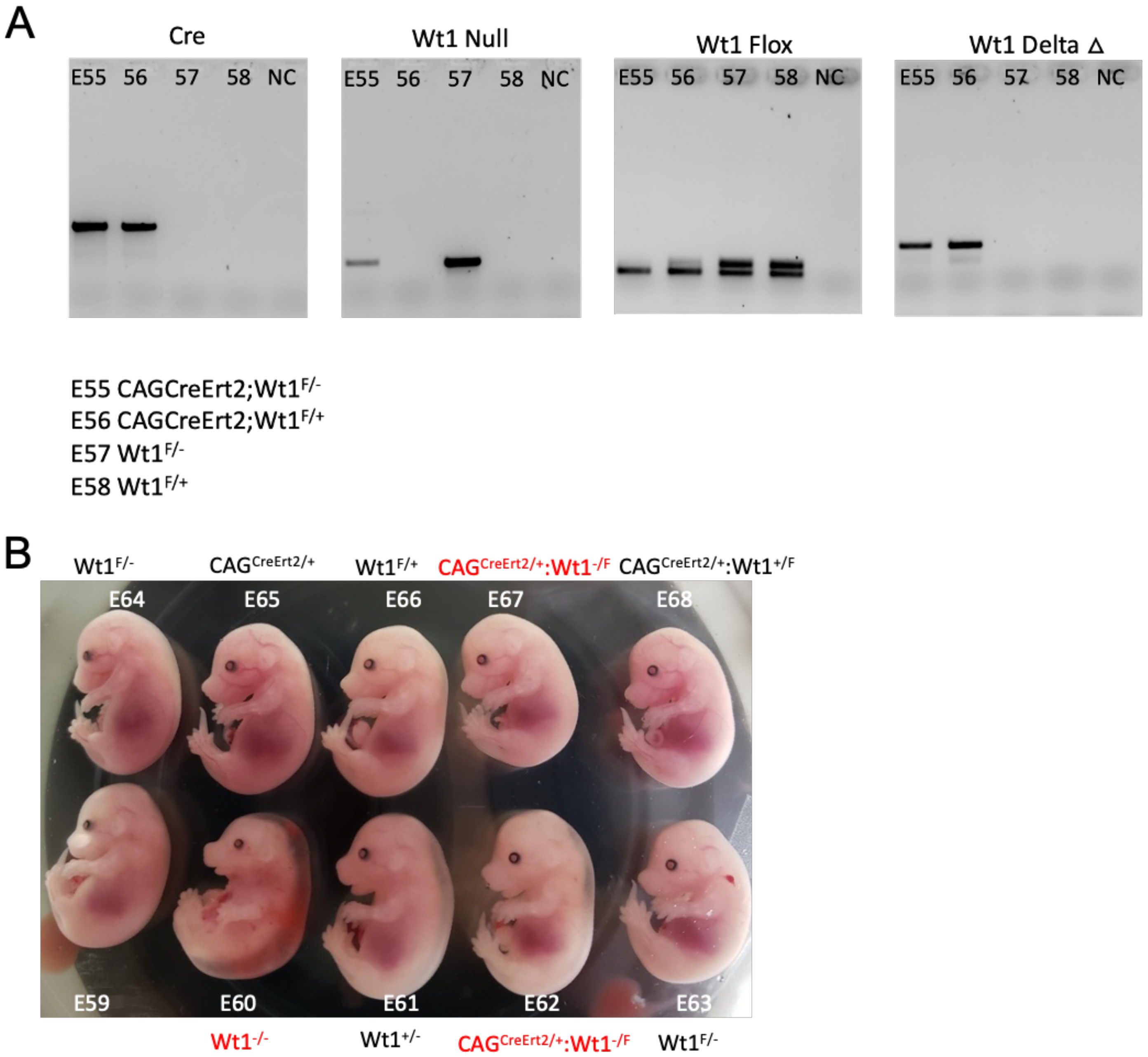

Supplementary Figure S8. Validation of Wt1 knockout (A) Confirmation of *Wt1* knockout. Murine embryos bearing both the *CAGG-Cre*$^{ER\text{-}TM}$ allele and the *Wt1*^{fl} allele were confirmed to exhibit detection of the *Wt1* delta allele, which confirms tamoxifen-inducible knockout of *Wt1*. NC – no DNA negative control. (B) Homozygous mice bearing a floxed *Wt1* allele (*Wt1*$^{fl/fl}$) were crossed with *Wt1*$^{+/-}$-*Cre-ER*^{TM} mice. Pregnant dams were treated with 5 mg/40g body weight intraperitoneal tamoxifen at E11.5 to activate Cre-mediated recombination and knock out *Wt1*. Embryos were harvested at E15.5. Embryos numbered E60, E67, and E62 are expected to exhibit lower levels of *Wt1*.
